# Widely distributed and regionally isolated! Drivers of genetic structure in *Gammarus fossarum* in a human-impacted landscape

**DOI:** 10.1186/s12862-016-0723-z

**Published:** 2016-07-29

**Authors:** Martina Weiss, Florian Leese

**Affiliations:** 1 Aquatic Ecosystem Research, University of Duisburg-Essen, Universitaetsstrasse 5, Essen, D-45141 Germany; 2 Centre for Water and Environmental Research (ZWU), University of Duisburg-Essen, Universitaetsstrasse 2, Essen, D-45141 Germany

**Keywords:** Realized dispersal, Environmental stressors, Freshwater organism, *Gammarus fossarum*, Gene flow, Genetic isolation

## Abstract

**Background:**

The actual connectivity between populations of freshwater organisms is largely determined by species biology, but is also influenced by many area- and site-specific factors, such as water pollution and habitat fragmentation. Therefore, the prediction of effective gene flow, even for well-studied organisms, is difficult. The amphipod crustacean *Gammarus fossarum* is a key invertebrate in freshwater ecosystems and contains many cryptic species. One of these species is the broadly distributed *G. fossarum* clade 11 (type B). In this study, we tested for factors driving the genetic structure of *G. fossarum* clade 11 in a human-impacted landscape at local and regional scales. To determine population structure, we analyzed the mitochondrial cytochrome c oxidase 1 (*CO1*) gene of 2,086 specimens from 54 sampling sites and microsatellite loci of 420 of these specimens from ten sites.

**Results:**

We detected strong overall genetic differentiation between populations at regional and local scales with both independent marker systems, often even within few kilometers. Interestingly, we observed only a weak correlation of genetic distances with geographic distances or catchment boundaries. Testing for factors explaining the observed population structure revealed, that it was mostly the colonization history, which has influenced the structure rather than any of the chosen environmental factors. Whereas the number of in-stream barriers did not explain population differentiation, the few large water reservoirs in the catchment likely act as dispersal barriers.

**Conclusions:**

We showed that populations of *Gammarus fossarum* clade 11 are strongly isolated even at local scales in the human-impacted region. The observed genetic structure was best explained by the effects of random genetic drift acting independently on isolated populations after historical colonization events. Genetic drift in isolated populations was probably further enhanced by anthropogenic impacts, as *G. fossarum* is sensitive to many anthropogenic stressors. These findings highlight the importance of small-scale genetic studies to determine barriers restricting gene flow to prevent further loss of genetic diversity and maintain intact freshwater ecosystems.

**Electronic supplementary material:**

The online version of this article (doi:10.1186/s12862-016-0723-z) contains supplementary material, which is available to authorized users.

## Background

Biogeographic studies revealed wide ranges for many freshwater invertebrate species [1]. This holds true in particular for species found in temperate and more northern latitudes, which had to recolonize habitats after glacial periods. Examples range from various insect species with terrestrial adult life stages to purely aquatic species, such as amphipod crustaceans and molluscs. Considering the typically wide ranges and high regional and local abundances, freshwater invertebrates are often regarded as frequent and long distance dispersers (e.g. [2, 3]). Over the past 15 years, however, the paradigm of wide ranges has been increasingly questioned, as molecular studies revealed the presence of morphologically cryptic species in many freshwater invertebrate taxa (e.g. [4–6]). These cryptic species often show rather small and allopatric ranges (e.g. [7, 8]), instead of the presumed broad distribution of the whole cryptic species complex.

One ecologically important freshwater taxon, which was formerly thought to be widely distributed in central and southeastern European streams, is the amphipod crustacean *Gammarus fossarum* KOCH, 1836. Several recent studies revealed an almost exponentially increasing number of overlooked species within the *G. fossarum* species complex with enhanced geographic sampling and improved sensitivity of molecular detection methods [9–11]. The highest species diversity, by far, within the *G. fossarum* species complex was found in the southeastern part of the range, where most of the newly discovered species were local endemics with narrow ranges [11]. However, the four central and western European species, in particular clade 11, still show broad distributions [10].

Generally, *G. fossarum* is mainly found in the upper reaches of streams and is sensitive to organic pollution [12, 13], high ammonium concentrations [14], a lack of oxygen, and acidification [15]. Owing to its high abundances and sensitivity to anthropogenic stressors, *G. fossarum* is often used in ecotoxicological studies (e.g. [16–18]). However, the precise cryptic species used in these experiments and whether a single or multiple species are used are rarely tested or reported. Validating species assignments prior to experiments is critically important, as studies explicitly investigating *G. fossarum* type A and B (here referred to as clade 12 and 11, after Weiss et al. [10]) revealed ecological differences between the species [19–21]. In further studies comparing these two species, clade 11 was found to be more tolerant against tested stressors [14, 22], occurred in areas with higher human impact [19] and was the better competitor in comparison with clade 12 [13], but it also showed higher infection rates for various parasites [23]. Additionally, in a direct comparison, populations of clade 11 were less differentiated across hundreds of kilometers than populations of clade 12 [24], but still significant differentiation within clade 11 was found on a regional scale. These findings agree well with the moderate genetic differentiation found in a broad geographic area for members of clade 11 (e.g. [10]). Even though these findings may indicate a relatively good dispersal ability for *G. fossarum* clade 11, it is difficult to predict actual dispersal rates, as they can be influenced by area- and site-specific environmental factors, like water chemistry, stream bed structure, land use and urbanization in the riverine environment, and fragmentation of streams by in-stream barriers, like dams or reservoirs (e.g. [25–27]). However, as understanding the patterns and mechanisms of dispersal and connectivity is crucial for predicting population resilience and long-term adaptability of a species [28], it is important to determine the actual dispersal rates. An already regularly applied approach for this purpose is the use of genetic markers to estimate effective gene flow between populations, i.e. successful dispersal leading to genetic exchange between populations (e.g. [3, 29, 30]).

In this study, we tested for factors driving the genetic structure of *G. fossarum* clade 11 in a human-impacted landscape at local and regional scales. To determine the population structure, we used two different genetic markers. For the main analyses, we used the barcoding fragment of the mitochondrial cytochrome c oxidase 1 (*CO1*) gene. We also examined nuclear microsatellite markers [31, 32] for a subset of populations to validate the *CO1* results. The study area was the Sauerland region, a low mountain range in North Rhine-Westphalia, Germany, which contains several small nature reserves, but is also used for agriculture, industry, forestry, and tourism. The hydrological structure of streams in the Sauerland region is strongly influenced by anthropogenic factors, such as in-stream barriers occurring approximately every 1,000 m [33]. Therefore, the region is characterized by high site heterogeneity in terms of ecological parameters as well as habitat fragmentation, making it an interesting area to study the impact of anthropogenic factors on the realized dispersal of aquatic invertebrates. To account for these factors, we characterized sampling sites based on several ecological parameters and combined dense small-scale sampling with broader regional sampling within a range of 85 km. Specifically, we tested the following hypotheses:

1. Populations of *Gammarus fossarum* clade 11 are genetically differentiated at the regional scale when considering the whole study area. In contrast differentiation is low at the local scale of few kilometers with evidence of gene flow between populations. These expectations are based on previous genetic analyses of *G. fossarum* species (e.g. [24, 34–36]).
2. Genetic variation is significantly partitioned after drainage basins at any spatial scale within the riverine network according to the Stream Hierarchy Model [37, 38], because *G. fossarum* is confined to aquatic habitats throughout its life cycle. Further, populations show patterns of isolation by distance (IBD) within catchments, especially when considering the waterway distance as this pattern was also found in other population genetic studies of *G. fossarum* clade 11 (e.g. [24]).

After testing the two hypotheses, we discuss the findings in order to identify possible drivers of population structure.

## Methods

### Study site and sampling

Specimens of *G. fossarum* were collected via kick-sampling at 54 sampling sites in 45 different streams in the Sauerland region (Germany). Animals were preserved in 96 % ethanol. The sampling sites were located in three major catchments (Ruhr: 48; Eder: 5; Lippe: 1). For the analyses, the Ruhr catchment was further divided into four sub-catchments, Lenne (12), Möhne (5), Volme (2), and Ruhr (29), where Ruhr means that the streams flow directly into the Ruhr and not via another main river, such as the Lenne (see Table 1 for details). To characterize the sampling sites, various parameters were measured (see Additional file 1). Directly determinable parameters were coordinates, sub-catchment association, altitude, distance to the spring, and if the sampling site was positioned in a small nature reserve. Land use types (estimated as percentages) in a buffer zone of 10 m wide and 1 km long upstream were determined in QGIS v. 2.8.2 [39] using the ATKIS land cover vector data [40]. The categories were conifer, broadleaf, and mixed forests, farmland, grassland, water bodies, and urban areas. The hydromorphological variables channel pattern (Main Parameter 1, MP1), longitudinal profile (MP2), and structure of water beds (MP3) were derived from the national hydromorphological survey ([41], described in [42]). Further, the ecological status according to the Water Framework directive [43] was determined for the sampled streams. Data for both hydromorphological variables and ecological status were provided by the federal state authority LANUV (Landesamt für Natur, Umwelt und Verbraucherschutz © Land NRW, Recklinghausen, http://www.lanuv.nrw.de). Chemical measurements were obtained from the ELWAS-web portal. Most of the official measuring points were not directly located at the sampling sites in this study, but 41 of these measuring points were located within a 5 km reach. The frequency with which the chemical values were measured differed among sites, varying from one to four years and one to seven times a year, but not all parameters were measured on all dates. To correct for these differences and variation within sampling sites, the average value for each chemical was calculated. The following chemical parameters were used for the analyses: calcium, iron, oxygen, chloride, ammonium, total organic carbon (TOC), total nitrogen, and pH (for details see Additional file 1).

**Table 1:** Sampling sites and number of studied *G. fossarum* specimens (n) in the Sauerland area

site	stream name	n	longitude (GK3)	latitude (GK3)	sub-catchment	genetic marker	sampling year
HSK10	Hannebecke	12	3458921.3	5685865.4	Ruhr	CO1	2014
HSK6b	Nierbach	26	3455373.8	5686737.7	Ruhr	CO1	2014
KL14	Bieberbach	60	3426167.2	5696528.9	Ruhr	CO1	2014
KL3	Waldbach	71	3432816.0	5681103.7	Ruhr	CO1	2014
KL9	Röhr 3	59	3434350.5	5682378.1	Ruhr	CO1	2014
LE	Leiße	53	3452683.7	5673900.3	Ruhr	CO1 & msat	2011 & 2013
ME	Medebach	18	3466979.7	5687670.3	Ruhr	CO1	2012
NG	Renau	46	3462376.4	5675757.3	Ruhr	CO1 & msat	2011 & 2013
NL	Namenlose	12	3464929.2	5675391.8	Ruhr	CO1	2011
PL1	Palme 1	25	3457449.9	5680115.8	Ruhr	CO1 & msat	2011
PL2	Palme 2	82	3458169.1	5676718.3	Ruhr	CO1	2011 & 2013
QB12	Elpe	36	3461411.5	5681943.7	Ruhr	CO1	2013
QB17	Ilpe 1	36	3445685.8	5678145.5	Ruhr	CO1	2013
QB22	Kleine Henne	58	3453172.3	5688126.2	Ruhr	CO1	2014
QB23	Ilpe 2	36	3447494.9	5677477.6	Ruhr	CO1	2014
QB24	Hengsbecker Bach	48	3442318.1	5677813.1	Ruhr	CO1	2014
QB27	Schürenbach	58	3446183.3	5688992.2	Ruhr	CO1	2014
QB29	Krähe	46	3425599.1	5681574.1	Ruhr	CO1	2014
RO1	Röhr 1	20	3434317.5	5679605.7	Ruhr	CO1	2012
RO2	Röhr 2	25	3431365.3	5686986.9	Ruhr	CO1	2012
RU3	Ruhr 3	52	3466754.4	5681626.1	Ruhr	CO1 & msat	2011 & 2013
RU4	Ruhr 4	7	3467531.0	5676847.1	Ruhr	CO1	2011
SB	Schlebornbach	58	3460025.7	5694421.7	Ruhr	CO1 & msat	2011 & 2013
VA1	Valme 1	20	3459155.3	5678350.2	Ruhr	CO1	2011
VR11	Refflingser Bach	47	3406403.2	5698078.2	Ruhr	CO1	2013
VR16	Elsebach	36	3404615.0	5697289.2	Ruhr	CO1	2013
VR17	Palme 3	44	3457758.0	5678654.0	Ruhr	CO1	2014
VR5	Valme 2	48	3458707.3	5683774.6	Ruhr	CO1	2013
VR7	Kelbke	48	3445543.3	5686405.8	Ruhr	CO1	2013
E01	Mühlenbach	14	3410770.9	5681556.6	Lenne (Ruhr)	CO1	2011
E02	Gleierbach	54	3453326.7	5672099.4	Lenne (Ruhr)	CO1 & msat	2011 & 2013
E04	Husberger Bach	29	3409316.4	5681432.9	Lenne (Ruhr)	CO1	2011
E06	Fretterbach	15	3434941.8	5674698.5	Lenne (Ruhr)	CO1 & msat	2011
E11	Elspe	22	3438640.3	5672688.0	Lenne (Ruhr)	CO1	2011
GB	Grüner Bach	29	3408046.7	5691018.5	Lenne (Ruhr)	CO1 & msat	2012
KL13	Schwarze Ahe	68	3410745.1	5675226.3	Lenne (Ruhr)	CO1	2014
KL15	Krummenau	59	3409854.3	5660639.6	Lenne (Ruhr)	CO1	2014
KL2	Worbscheider Bach	57	3417114.1	5663534.7	Lenne (Ruhr)	CO1	2014
NB	Nimmer Bach	37	3400329.5	5688143.1	Lenne (Ruhr)	CO1	2011 & 2012
SO	Sorpe 1	14	3458195.6	5673861.6	Lenne (Ruhr)	CO1	2011
VR2	Sorpe 2	56	3460431.0	5673996.4	Lenne (Ruhr)	CO1	2013
AA	Aa	17	3467971.2	5696423.1	Möhne (Ruhr)	CO1	2011
GS	Große Schmalenau	30	3441171.5	5701881.2	Möhne (Ruhr)	CO1	2012
KL6	N.N.	60	3465023.5	5697739.9	Möhne (Ruhr)	CO1	2014
LO	Lörmecke	30	3458555.9	5702556.6	Möhne (Ruhr)	CO1	2011
QB10	Hirschberger Bach	27	3453285.9	5701355.8	Möhne (Ruhr)	CO1	2013
VR12	Ennepe	48	3395008.0	5671675.1	Volme (Ruhr)	CO1	2013
VR23	Epscheider Bach	56	3393785.4	5683027.4	Volme (Ruhr)	CO1	2014
BB	Bremke-Bach	29	3466644.6	5668828.9	Eder	CO1	2011
HB1	Hallebach 1	16	3478675.0	5677590.1	Eder	CO1	2011
HB2	Hallebach 2	20	3474568.9	5675458.9	Eder	CO1	2011
ND	Neerdar	24	3478218.4	5682425.6	Eder	CO1	2011
NH	Nuhne	58	3467984.5	5671089.2	Eder	CO1 & msat	2011 & 2013
AL	Alme	30	3473839.9	5701645.1	Lippe	CO1 & msat	2011

*G. fossarum* sampling was conducted in 2011, 2012, 2013, and 2014 (Table 1). Samples from six sites in 2011 were provided by Maria Gies and Martin Sondermann (University Duisburg-Essen). To examine changes in the genetic structure of populations over time, seven 2011 sampling sites were resampled in 2013 and one 2011 site was sampled again in 2012 (Table 1). Additional samples from 2013 and 2014 were originally obtained for another study in which a stream was sampled at four sites every 200 m with an in-stream barrier between separating sites two and three. At seven sampling sites in 2014, a similar sampling scheme was used for sewage plants, and, at two sampling sites, similar schemes were used for mining sites. Since no differences in haplotype frequencies were found between those sites (*F*_ST_ values were not significant, data not shown), the samples were merged into a single sampling site for this study.

### Sequencing and genotyping

DNA was extracted from the pereopods of 2,086 specimens using a modified salt-extraction protocol [44] (see Additional file 2 for details of used protocol). For all specimens, a fragment of the mitochondrial barcoding gene *CO1* was amplified with the standard primers HCO2198 and LCO1490 [45] using the following PCR protocol: 1× PCR buffer, 0.2 mM dNTPs, 0.5 μM each primer, 0.5 μl of DNA template, 0.025 U/μl HotMaster Taq (5 PRIME GmbH, Hamburg, Germany), filled to 15 μl with sterile H_2_O. The PCR settings for *CO1* amplification were as follows: initial denaturation at 94 °C for 2 min; 34 cycles of denaturation at 94 °C for 20 s, annealing at 46 °C for 30 s, and extension at 65 °C for 60 s; final extension at 65 °C for 5 min. The PCR products (9 μl) were purified using 0.5 μl of ExoI (20 U/μl) and 1 μl of FastAP (1 U/μl, both enzymes; Thermo Fisher Scientific, Schwerte, Germany). The reaction was incubated for 25 min at 37 °C followed by an inactivation step at 85 °C for 15 min. Purified PCR products were bidirectionally sequenced by GATC-Biotech (Konstanz, Germany).

For 420 specimens from ten sampling sites (Table 1), eight microsatellite loci were additionally amplified: Gam 2, Gam 14 [31], Gamfos 10, Gamfos 13, Gamfos 18, Gamfos 22, Gamfos 27, and Gamfos 28 [32]. For each primer pair, the originally published sequence was adapted to our analysis system by adding a universal M13 tail (5′-CAC GAC GTT CTA AAA-3′) to the 5′ ends of the forward primers for primers developed by Danancher et al. [31] and to the reverse primers for those developed by Westram et al. [32]. The amplification of Gam 2, Gam 14, Gamfos 13, Gamfos 27, and Gamfos 28 was performed using the following protocol: 1× PCR buffer, 0.2 mM dNTPs, 0.2 μM sequence-specific untailed primer, 0.05 μM sequence-specific tailed primer, 0.2 μM fluorescently labelled universal M13 primer, 5 % dimethyl sulfoxide (DMSO), 0.5–1 μl of DNA template, 0.025 U/μl HotMaster Taq (5 PRIME GmbH, Hamburg, Germany), filled to 15 μl with sterile H_2_O. For Gamfos 10, 18, and 22, betaine was used instead of DMSO. PCR settings for the amplification of Gam2, Gam14, Gamfos 13, Gamfos 18, Gamfos 22, and Gamfos 28 were as follows: initial denaturation at 94 °C for 2 min; 36 cycles of denaturation at 94 °C for 20 s, annealing at 60 °C for 30 s, and extension at 65 °C for 30 s; final extension at 65 °C for 45 min. For Gamfos 10 and Gamfos 27, an annealing temperature of 54 °C was used.

Allele sizes were determined using polyacrylamide gel electrophoresis on a LI-COR® 4300 DNA Analyzer with the software Saga^GT^ (LI-COR Biosciences, Bad Homburg, Germany). For Gam 2, the alleles could not be scored because too many stutter bands occurred over a range of 90 base pairs.

### Sequence data analysis

Sequences were assembled and edited in Geneious 8.1.2 (http://www.geneious.com, [46]), and a multiple sequence alignment was computed with MAFFT [47] as implemented in Geneious (automatic algorithm selection, default settings). Genetic diversity was calculated as haplotype diversity using Arlequin 3.5 [48]. A minimum spanning network was calculated using Arlequin and visualized using HapStar 0.7 [49]. To show the position of the haplotypes in a broader phylogenetic context nine sequences from five different clades (3, 10, 11, 12 and 13), which have been used in the study of Weiss et al. [10] (GenBank Accession numbers: JN900490, KF521805, KF521817, KF521822, KF521828, KF521829, KF521832, KF521833, KF521835), were added to a dataset of the main haplotypes of this study and a neighbor-joining Tree was calculated with MEGA6 [50], with evolutionary distances computed using the Kimura 2-parameter method. To test for population differentiation, pairwise *F*_ST_ values between populations from different sampling sites were calculated using Arlequin. Negative values were set to zero. The significance levels for this and all subsequent *F*_ST_ calculations were adjusted to account for multiple testing using the false discovery rate control (FDR, [51]). Additionally, *F*_ST_ values were calculated using data from multiple years for populations originating from the same stream to test if haplotype frequencies changed significantly over time.

### Microsatellite data analysis

Using the program MICRO-CHECKER 2.2.3 [52], our data set was checked for the occurrence of allelic drop out, stutter bands, and null alleles and null allele frequencies were calculated using the Dempster method [53]. All loci in all populations were tested for deviations from Hardy–Weinberg equilibrium (HWE) and from linkage equilibrium using Arlequin. To estimate genetic diversity, allelic richness was calculated with the program HP-Rare 1.0 [54] using rarefaction to correct for differences in sample sizes.

Genetic distances between populations were calculated as pairwise *F*_ST_ values in Arlequin. To correct for null alleles, FreeNA [55] was used, in which the ENA correction was implemented. To test if the allele frequencies in the populations changed over time, *F*_ST_ values were also calculated between populations originating from the same stream, but sampled in different years.

### Determination of drivers of genetic differentiation (*CO1* data)

Different landscape genetic approaches were used to identify drivers of genetic differentiation in the study area. First, to test if the distribution of genetic variance can be explained by the partitioning of populations into the six sub-catchments, AMOVA (analysis of molecular variance, [56]) was implemented in Arlequin. To test if genetic distances were correlated with geographic distances (isolation by distance, IBD), Mantel tests were performed in Arlequin. Pairwise *F*_ST_ values were used as a measure of genetic distance, and either straight-line or waterway distances were used for geographic distance. Straight-line distances were calculated for all population pairs and waterway distances for populations within the Ruhr catchment (*n* = 48) using QGIS v. 2.8.2 [39] and the map containing the streams provided by the federal state authority LANUV (Gewässerstationierungskarte des Landes NRW © LANUV NRW (2013)). To test if in-stream barriers influenced the connectivity between populations, another Mantel test was conducted with 21 populations of the Ruhr sub-catchment using pairwise *F*_ST_ values and the number of in-stream barriers between population pairs per km waterway distance, first for barriers >0.5 m and then for barriers >1 m. The number of in-stream barriers was calculated from the QUIS database [57]. Barriers were mostly weirs and barrages, often classified as only partly or not at all passable for the aquatic fauna in the ELWAS-web portal. To investigate if spatial factors other than geographic distance or catchment assignment shape genetic structure and to infer the number of populations, spatial Bayesian clustering models were implemented in the R package GENELAND, v4.0.5 ([58, 59], R version 3.2.2, [60]). The number of populations K was allowed to vary between 1 and 20. For six independent runs, 1,000,000 MCMC iterations were calculated, sampling every 100 steps. The maximum number of nuclei in the Poisson-Voronoi tessellation was set to 6,300. For post-processing, a burn-in of 1,500 iterations was used and the pixels along the *X*-axis were set to 300 and along the *Y*-axis to 150 according to the ratio of the sampling area. A second AMOVA implemented in Arlequin using the groups detected in GENELAND was used to analyze if this clustering better reflects the population structure than the sub-catchments.

To test which factors determine the association of the individual populations to the GENELAND groups, a discriminant analysis was performed in IBM SPSS v. 23. For this analysis, the environmental variables obtained to characterize sampling sites were independent variables and the main GENELAND groups (consisting of >3 sites) were dependent variables. Additional to the environmental variables, also the geographic position of the sampling sites, represented by their coordinates, was used as a variable to include a proxy for the possible influence of colonization history on genetic structure. To standardize the predictor variables, z-scores were calculated in SPSS prior to the discriminant analysis. Further, multicollinearity between the 25 environmental variables was tested by calculating the variance inflation factors (VIFs) for each variable using the R package “usdm” [61]. Collinearity between variables was assumed for VIFs >10. After calculating the VIFs, the variables, which were highly collinear, were stepwise excluded from the analysis until all values were > 10. Therefore, the following discriminant analysis was conducted with a reduced set of variables. In the discriminant analysis, the stepwise Wilks lambda method was used to select predictors.

## Results

**Fig. 1:**
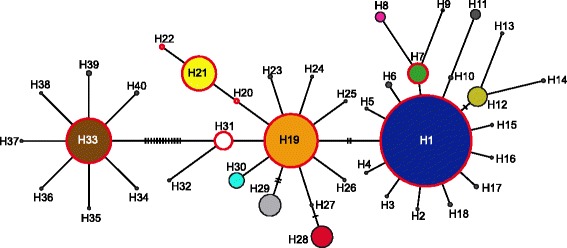
Minimum spanning network created from *CO1* sequences. Circles represent different haplotypes and their dimensions are scaled based on the number of sequences, which are given in Table 1. Vertical lines represent missing or unsampled haplotypes. Red edges of circles indicate that these haplotypes were found at different sampling sites, while black edges indicate private haplotypes. Haplotypes are colored similar to Fig. 2a

We generated *CO1* sequences for 2,086 *G. fossarum* clade 11 specimens from 54 sampling sites. In the 557 bp alignment, we detected 52 variable sites, of which 12 were non-synonymous substitutions. Specimens clustered into 40 distinct haplotypes (H1–H40), of which ten had a frequency of over 1 % (H1, H7, H12, H19, H21, H28–31, and H33) and were found in 97.2 % of all specimens (Additional file 3). We observed the most common haplotype, H1, in 38 populations and 48.8 % of sequences. Other common haplotypes were H19 (19 populations; 16.7 % of sequences), H21 (8; 6.8 %), and H33 (6; 11.6 %), and we found all four haplotypes in at least three sub-catchments. Each of these common haplotypes had further derived ones, which were mostly differentiated by a single mutation, visible in the haplotype network (Fig. 1). H33 and the surrounding haplotypes H34 to H40 were differentiated by at least 14 substitutions (distance between H33 and H31) from all other haplotypes. In the neighbor-joining tree (Additional file 4), all haplotypes clustered together with sequences belonging to Clade 11. As already visible in the network, this clade was divided into two sub-clusters (one containing H33 and the second all other main haplotypes), but the distance between the two sub-clusters was shallow as compared to distances between the different clades.

We only detected eight haplotypes that were shared between at least two populations (H1, H7, H19–22, H31, and H33) and we observed private haplotypes in 20 populations. At two sampling sites, i.e., KL15 (*n* = 59) and KL2 (*n* = 57), populations consisted exclusively of one private haplotype each. Overall, we detected between one and six haplotypes per population, with an average of two haplotypes, and a haplotype diversity of between 0.00 and 0.68, with an average of 0.16 (Additional file 3). The nucleotide diversity ranged from 0.000 to 0.136 with an average of 0.048 (Additional file 3). In seven of the eight populations for which we compared haplotype frequencies among years, we did not observe significant differences. The only exception was population NH, where, in the second sampling year, only the main haplotype of the population (H21) was rediscovered together with a new haplotype (H20), resulting in a small, but significant, *F*_ST_ value.

Of the seven microsatellites analyzed to complement the *CO1* data set, only four were polymorphic in the studied populations (Gamfos 10, 13, 18, and 28). Three of the ten populations were also monomorphic for the same allele at Gamfos 13. We observed between 4 and 23 alleles in all populations at the different loci. We did not detect significant linkage between loci considering all populations. We found evidence for null alleles for three of the loci in different populations. The null allele frequencies, number of alleles, and observed and expected heterozygosity (H_O_ and H_E_) are given in Additional file 5. Null allele frequencies ranged from 0.00 to 0.29. We observed deviations from HWE in all populations for at least one locus (see Additional file 5). As a measure of genetic diversity, we estimated allelic richness using rarefaction for a minimum sample size of 15 diploid individuals. We observed an average allelic richness over all loci of between 2.5 and 5.3 and we detected private alleles in six of the populations (Additional file 5). In two of the populations sampled twice in different years (NH and E02), the allele frequencies changed over time, resulting in small, but significant, *F*_ST_ values.

### Regional and local differentiation

**Fig. 2:**
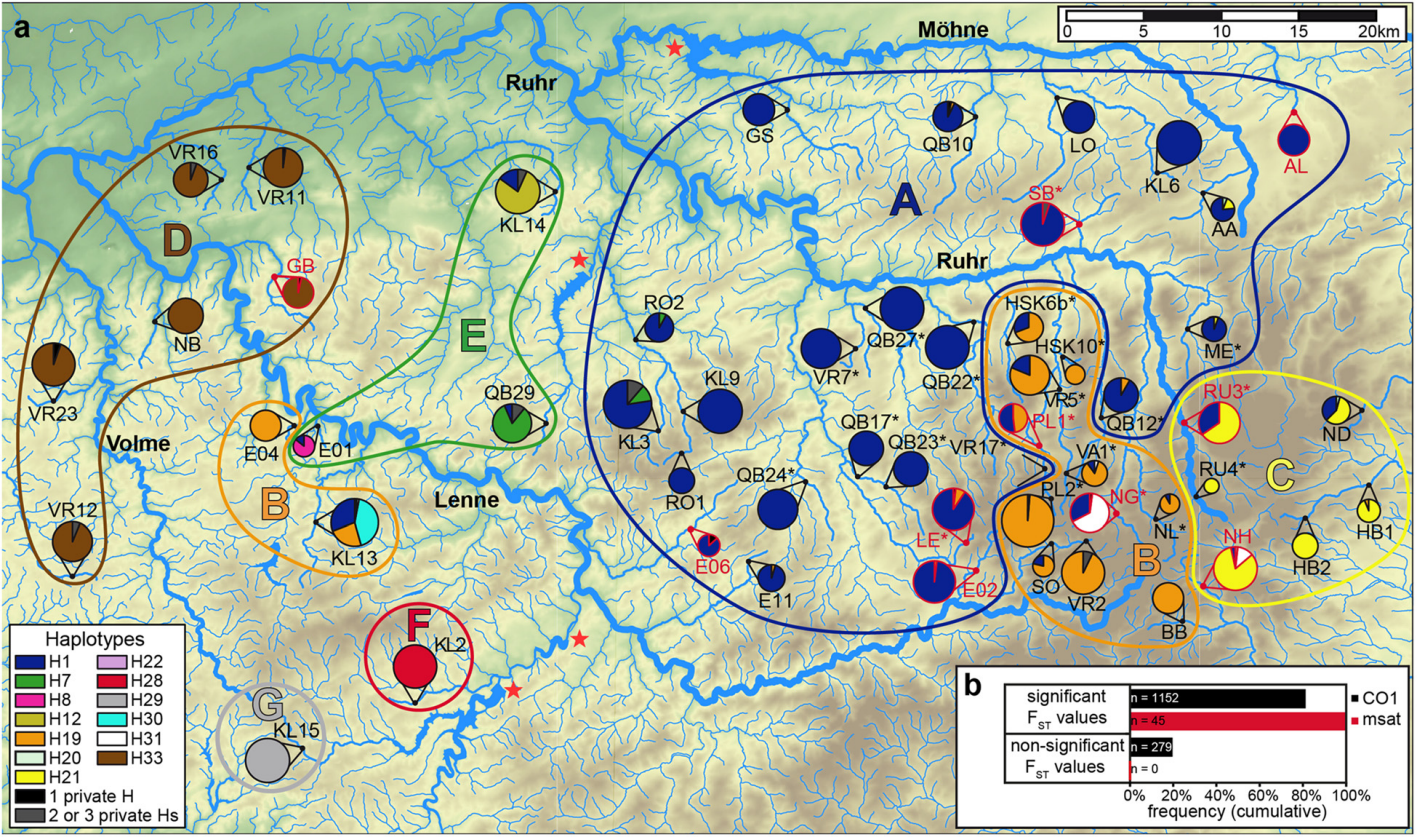
**a** *CO1* haplotype map showing the haplotype composition for *G. fossarum* at different sampling sites in the Sauerland region. The sizes of haplotype pie charts are scaled according to the numbers of sequences per site, which are given in Table 1 together with the sub-catchment association of sampling sites. Red stars indicate water reservoirs. Red highlighted sampling sites indicate that microsatellites were analyzed at these sites. All private low-frequency haplotypes are colored in black, or in gray if more than one private haplotype was found at the respective site. Colored contour lines illustrate GENELAND groups, named A–H. **b** Bar chart showing the frequency of significant and non-significant *F*_ST_ values for microsatellites (msat) and *CO1* sequences

The *CO1* haplotype composition at the sampling sites differed strongly in many cases on a regional as well as on a local scale (Fig. 2a), resulting in an overall high differentiation (mean *F*_ST_ = 0.61), and 81 % of all pairwise *F*_ST_ values were significant (1,152 out of 1,431 comparisons; Fig. 2b and Additional file 6). Considering only the subset of populations in which microsatellites were analyzed, 82 % of the *F*_ST_ values indicated significant population differentiation. All of the populations in this subset were significantly differentiated from each other when analyzing microsatellite loci (Fig. 2b, Additional file 7), with *F*_ST_ values ranging between 0.07 and 0.57. When estimating *F*_ST_ values with FREENA using the ENA method to correct for null alleles, they were slightly lower in most cases, ranging from 0.06 to 0.55 (Additional file 7). Altogether, we detected strong signatures of local and regional isolation of *G. fossarum* populations using both data sets*.*

### Drivers of local isolation

**Fig. 3:**
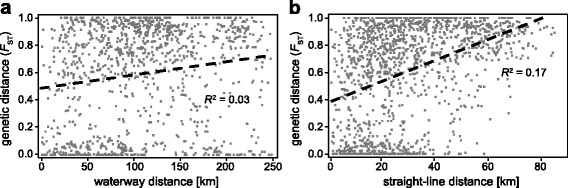
**a** Correlation between pairwise genetic and waterway distances for sampling sites of the whole Ruhr catchment. **b** Correlation between pairwise genetic and straight-line distances for all sampling sites

The AMOVA using six sub-catchments as groups revealed significant population differentiation between sub-catchments, with 19.6 % of the variation partitioned between groups. However, 59.8 % of the variation was between populations within sub-catchments (Table 2). Mantel tests revealed small but significant correlations between genetic distance (pairwise *F*_ST_) and geographic distance. Here, the fit was better using straight-line distances (*R*^2^ = 0.17, *p* = 0.000) than waterway distances (*R*^2^ = 0.03, *p* = 0.010, see Fig. 3). We did not detect correlations between genetic distance and the number of barriers per km between a subset of 21 sampling sites, for two barrier size thresholds (barriers >0.5 m: *R*^2^ = 0.0003, *p* = 0.433; barriers >1 m: *R*^2^ = 0.0007, *p* = 0.408).

**Table 2:** Results of AMOVA analysis according to 1) sub-catchments and 2) GENELAND groups

		Between sub-catchments			Between GENELAND groups		
Source of variation		d.f.	% variation	Fixation index	d.f.	% variation	Fixation index
between groups	*F*_*CT*_	5	19.6	**0.20**	6	72.1	**0.72**
between populations within groups	*F*_*SC*_	48	59.8	**0.74**	47	10.0	**0.36**
within populations	*F*_*ST*_	2032	20.6	**0.79**	2032	17.9	**0.82**

d.f. = degrees of freedom; bold values for Fixation index indicate significant population differentiation

Other major barriers located between some of the sampling sites were water reservoirs (see Fig. 2a). One such water reservoir, the Bigge reservoir, separated populations KL2 and KL15, and a second smaller one (Ahauser reservoir) separated both sites from all other sampling sites; both populations consisted only of one private haplotype each (H28 and H29, resp., see Fig. 2a). Another reservoir (Sorpe reservoir) separated QB29 from the other sampling sites. Between QB29 and the next sampled population (RO2), two haplotypes were shared, but the haplotype composition was significantly different. The population GS of the Möhne sub-catchment was separated by the Möhne reservoir from the other populations of this catchment. This reservoir also separated the whole sub-catchment from the Ruhr catchment. In both the GS population and some populations from the Ruhr and Möhne catchment (e.g. KL9, VR7, and LO), we only detected haplotype H1, and we did not detect a barrier effect of the Möhne reservoir based on the *CO1* sequence analyses.

To infer the population structure more directly, without manually assigned assumptions regarding affiliations to catchment areas, we performed a clustering analysis with GENELAND. In this analysis, we observed seven distinct groups, termed A–G (letters in circles in Fig. 2a). The biggest group was group A, with 24 populations containing mostly or exclusively haplotype H1. The second biggest group, dominated by H19, was group B with 13 populations. In most of these populations, we also detected H1, although at minor frequencies. This group was geographically split into two subgroups, with two populations located in the western area, and most populations in the east, with no visible connection between the two subgroups. Groups C and D were the easternmost and westernmost groups, both consisting of six populations, dominated by H21 and H33, respectively. While populations of group C shared haplotypes with groups A and B, all haplotypes of group D (H33 to H40) were exclusively found in that group. All but one population of group D contained additional private haplotypes, aside from the main haplotype. Group E consisted of three populations that shared H1 at a minor frequency, but had different dominant haplotypes, which were all derived from H1 (Fig. 1). Groups A to E all contained populations from at least two sub-catchments. The last two groups F and G each consisted only of one population (KL2 and KL15) and contained exclusively one private haplotype (H28 and H29, respectively). Both haplotypes were three mutations apart from H19 and six from each other. Using the GENELAND groups in an AMOVA, we found that this clustering reflects the population structure better than the sub-catchment structuring, as 71.1 % (instead of 19.6 %) of the variation was between groups and only 10 % (instead of 59.8 %) was among populations within groups (Table 2).

To test if other environmental factors determine the observed population structure, we gathered data for 25 environmental variables (Additional file 1). As groups F and G only consisted of single sites and populations of group E were highly differentiated from each other, we only conducted the following analyses for the four main groups, A–D. Testing for multicollinearity revealed that some of the variables were highly correlated. We therefore excluded the following variables for subsequent analyses: altitude, MP3, grassland, total nitrogen, and pH, resulting in 20 variables for the discriminant analysis. The stepwise discriminant analyses revealed that the most useful variable for predicting assignments to the GENELAND groups was longitude; all other variables were not included in the model. With the resulting model, 65 % of the populations clustered correctly to the four groups, with differences in prediction performance between groups (see Additional file 8). While all populations of groups C and D clustered correctly, this was the case for only 61.5 % of group B and 50.0 % of group A populations, as these two groups had a broader geographic range with overlapping longitudinal values.

## Discussion

We analyzed a large number of specimens and populations of the freshwater crustacean *G. fossarum* clade 11 in the anthropogenically heavily impacted Sauerland region to identify factors that influence population structure and limit dispersal in this species.

Our first hypothesis was that populations are genetically differentiated when considering the regional scale of the whole study area, whereas the differentiation is low at the scale of few kilometers. In agreement with the first expectation of this hypothesis we detected strong regional differentiation in the Sauerland area, especially between the eastern and westernmost populations. The westernmost populations (GENELAND group D) contained only haplotypes (H33–H40) separated by at least 14 substitutions from the other haplotypes (H1–H32), but we did not detect this high degree of differentiation using microsatellites. A similar pattern of east-west differentiation was observed in the stonefly *Dinocras cephalotes* in the same study area [62]. Specifically, two highly divergent haplotype groups were found for the *CO1* gene. However, contrary to our findings, Elbrecht *et al*. [62] found haplotypes of these groups to be shared across populations of these groups and also detected ongoing gene flow using nuclear genes. As the stonefly has a terrestrial and more mobile life stage, these contrasting patterns may be explained by differences in mobility. The observed divergence between the two *G. fossarum* clade 11 groups (east-west) is likely the result of independent historic isolation in eastern and western refugia, as suggested by Elbrecht et al. (2014) for the stonefly species. Even though *G. fossarum* populations belonging to the two divergent groups seem to be isolated, it is likely that they can still interbreed when in contact. This is indicated by the low divergence (between 2.51 and 3.95 %) in comparison to previous estimates of divergence between cryptic species of *G. fossarum* [10], also visible in the phylogenetic tree, when comparing intra- and inter-clade divergence*.* Further, Lagrue et al. [63] found reproductive isolation between *G. fossarum* clades only when *CO1* sequence divergence was greater than 4 %.

We did, however, not only find strong differentiation between populations at a regional scale, but contrary to our hypothesis also at a local scale. Actually, most of the populations were highly isolated from each other (80 % of pairwise *CO1 F*_ST_ values were significant), sometimes even within 2 km in the same stream. The strong isolation is further supported by the pattern that many populations contained private haplotypes, which mostly differed by only a single mutation from the main haplotype of the respective population. This indicates long-term isolation because independent mutations were able to accumulate in the populations. In contrast to the overall strong isolation, we found little or no differentiation between some of the populations separated by over 40 km straight-line distances and even higher river distances (especially within GENELAND groups A and D) for the *CO1* gene. However, when analyzing a subset of these populations based on the more rapidly evolving microsatellites, we detected strong differentiation between all populations. Therefore, the subtle differentiation found between populations of group A is most likely not caused by ongoing gene flow; instead, low genetic variation is likely due to bottlenecks and the effects of genetic drift [6, 64].

In their study, Westram et al. [24] concluded that differences in population structure between clade 11 and 12 could hint at interspecific differences in dispersal ability, life history or population size. The authors discussed that differences in differentiation levels reflect more likely species specific rather than being driven by geographic effects. However, the strong local differentiation we found in clade 11 resembled much more the pattern described for clade 12 than that of clade 11 [24, 36]. The discrepancies observed between the data of Westram et al. [24] and our study suggest that geographic effects can strongly impact on the differentiation of populations and with that probably on the realized dispersal of populations within a species.

Based on the SHM [37] our second hypothesis was that the populations are structured according to catchment boundaries and show an IBD pattern within catchments. The structuring of populations according to catchments has been shown for other crustaceans [65, 66] and an IBD pattern has been detected in *G. fossarum* [12, 34, 36], specifically in clade 11 [24]. Studying variance partitioning and using the sub-catchment boundaries in an AMOVA, we detected significant partitioning of genetic variance, but most of the variance (59.8 %) was found between populations within groups, indicating that populations were not primarily structured by sub-catchments, but were locally isolated within sub-catchments. We found a similar pattern when analyzing the correlation between genetic and geographic distances, as the correlation was significant, but very weak for both geographic distance metrics (straight-line and waterway distance), especially for waterway distances. The weak signature of sub-catchment-based structuring and the stronger correlation between genetic and straight-line distances in comparison to waterway distances indicate that processes other than low dispersal within streams are relevant to the genetic structure within this species. This inference is also supported by the GENELAND analysis, where groups A–E contained populations from at least two sub-catchments each and two populations in group B were geographically distant to all other populations of this group. Evidence for overland dispersal in aquatic invertebrates, including *G. fossarum*, was reported previously (e.g. [36, 67, 68]), and it is assumed to occur by transport via vectors like birds [69–71], large mammals [72, 73], or humans [13]. As some of the springs of the different sub-catchments in our study area are only separated by a few hundred meters and not by mountain ranges, overland dispersal is likely possible. However, as pairwise differentiation was high in general, overland dispersal seems to be more important for rare colonization events on evolutionary time scales rather than for recurrent dispersal at ecological time scales within generations. However, we did not expect the low correlation between waterway and genetic distances. A lack of IBD in other aquatic invertebrates is often attributed to particularly weak (e.g. [6, 74–76]) or strong dispersal abilities (e.g. [27, 76–78]). These results may also be explained by the presence of strong dispersal barriers between populations [79–81]. The slight IBD pattern we found in our study was mainly caused by the strong differentiation between the western- and easternmost populations, as they were separated by the greatest geographic distances and did not share any haplotypes. Otherwise, for many pairwise comparisons, we detected high genetic differentiation at small geographic distances and no differentiation over long distances. As we found strong population differentiation between populations, indicated by high and significant *F*_*ST*_ values, the lack of IBD cannot be caused by too strong realized dispersal. However, previous studies found IBD in different areas for *G. fossarum* (e.g. [24]); accordingly, the dispersal ability should generally be sufficient to generate this pattern even though the realized dispersal appears to be very limited here. Therefore, it seems likely that other barriers to gene flow exist. These barriers could either be direct, like dams, weirs, or water reservoirs, or indirect if the conditions in connecting areas are unfavorable due to, for example, anthropogenic land use, organic pollution, acidification, large connecting rivers, or strong competition (e.g. [20, 27, 36, 68, 74, 76, 82, 83]). To analyze the influence of direct in-stream barriers, we used a subset of populations to determine if the number of barriers was correlated with the genetic distance between populations. Based on Mantel tests conducted for barriers of >0.5 m and >1 m, we did not observe a correlation with genetic distance, indicating that population isolation did not simply reflect the number of barriers. However, the influence of these barriers on realized dispersal could not be determined using the current marker and sampling scheme. To infer the influence of in-stream barriers more directly, individual barriers should be tested using more rapidly evolving genetic markers. Some of the sampling sites were separated from each other by water reservoirs, and, with one exception, the separated populations were significantly differentiated from all other analyzed populations, indicating a strong barrier effect of these water reservoirs. One exception at a first glance was in the Möhne sub-catchment, where populations were dominated by or even consisted exclusively of H1. However, as discussed previously, the diversity for the *CO1* gene in this region was too low to detect barrier effects. Therefore, we conclude that in-stream barriers, especially water reservoirs, can have a substantial impact on dispersal, yet they do not explain the general population structure observed in this study. With the exception of the water reservoirs that separate the clearly differentiated groups F and G from all other populations there were no obvious weir- or dam-related boundaries separating the groups.

In view of the tested parameters that cannot explain population structure sufficiently the question arises, which other factors ultimately underlie the apparent population structure. To determine if the structure was more influenced by colonization history (represented by the geographic position of the population) or by environmental factors that may differ between population groups, we conducted a discriminant analysis for the four main groups including variables for both possible drivers. The best predictor for the assignment of populations to the four GENELAND groups was longitude, indicating that the location of each site from east to west determines the affiliation to the *CO1* clusters. This clearly suggests that the observed population structure primarily reflects colonization history. If colonization events occur rarely and are initiated by relatively few individuals, this can lead to strong founder effects; genetic drift in small populations can lead to a strong loss of genetic diversity [64, 84]. The maintenance of this structure over time is probably promoted by a high degree of population isolation, leading to small effective population sizes and thereby enhancing the effect of random genetic drift. As *G. fossarum* is sensitive to many anthropogenic stressors, like organic pollution, a lack of oxygen, acidification [15], and high ammonium concentrations [14], isolated populations likely underwent drastic population declines over time, resulting in the loss of genetic diversity in this anthropogenically impacted area. Even though we could not identify a single anthropogenic factor influencing the population structure, we found populations to be highly isolated on local scales, clearly indicating that there are barriers to gene flow, which were not detectable with the methods used here.

## Conclusion

In this study we found a considerably higher differentiation between populations of *G. fossarum* clade 11 in the human-impacted Sauerland area than was expected based on previous genetic analyses. The strong isolation was supported by two independent molecular marker systems, indicating that the realized dispersal was low in the study area. Also contrary to our hypotheses we found only a slight isolation by distance (IBD) pattern and structuring of populations according to river catchments but rather a strong geographic pattern (east-west differentiation). In view of published data, the dispersal ability of *G. fossarum* clade 11 specimens should be sufficient enough to create an IBD pattern. In the absence of this we conclude that there are barriers preventing gene flow partly, even between neighboring populations. Despite the likely effect of larger reservoirs on connectivity, we could not determine specific anthropogenic factors that directly influence the population structure. In fact, it was best predicted by the independent action of genetic drift at local sites after initial colonization. These effects are likely enhanced by the multitude of anthropogenic stressors, because *G. fossarum* is sensitive to many anthropogenic stressors. As *G. fossarum* clade 11 is widely distributed and is frequently found in the Sauerland area we conclude that its colonization ability over long time scales is good. The same holds likely true for its ability to establish new populations based on few colonizers, since long distance dispersal by animal vectors is unlikely to occur frequently [3]. Even though we could not determine specific anthropogenic factors hindering gene flow, it is likely that the dispersal is influenced by these factors because of the exceptionally strong differentiation we found here in contrast to expectations based the findings of an earlier study (Westram et al. [24] and the much broader distribution of clade 11 in comparison with the other *G. fossarum* clades. These findings highlight the importance to take regional factors into account when predicting the dispersal ability of species. Further more research is needed to determine the most important barriers restricting gene flow between populations to prevent further losses of genetic diversity and maintain an intact ecosystem.

## Abbreviations

AMOVA, Analysis of molecular variance; *CO1*, cytochrome c oxidase 1 gene; FDR, False discovery rate; *F*_*ST*_, Fixation index; HWE, Hardy-Weinberg equilibrium; IBD, isolation by distance; SHM, Stream Hierarchy Model; VIF, variance inflation factor

## Additional files

Additional file 1: Ecological parameters for individual sampling sites. Group indicates the GENELAND group association of the sampling sites. Site is the abbreviation for each sampling site, and geographic coordinates are given in the Gauss–Krueger coordinate system. NA: no data were available for this site. (PDF 66 kb) Additional file 2: DNA salt-extraction protocol, modified from Sunnucks & Hales [44]. (PDF 51 kb) Additional file 3:
*CO1* haplotype information for each sampling site. Group indicates the GENELAND group association of the sampling sites, site is the abbreviation for the sampling site, and n refers to number of analyzed specimens per site. # H gives the total number of haplotypes at a site and private H is the number of private haplotypes. H diversity is the haplotype diversity and H1 to H40 represent the different haplotypes. (PDF 67 kb) Additional file 4:
**a:** Sampling locations of specimens used in the phylogeny. Symbols in the map correspond with symbols in **b** to indicate sampling locations of used sequences; the red square indicates the Sauerland area in which the specimens belonging to the main haplotypes were sampled. **b:** Neighbor-joining tree of main haplotypes and additional sequences belonging to clades 3, 10, 11, 12 and 13 (sensu Weiss et al. [10]). The tree is drawn to scale, with branch lengths (next to the branches) in the same units as those of the evolutionary distances used to infer the phylogenetic tree (K2P method). (PDF 2114 kb) Additional file 5: Microsatellite loci information for the different sampling sites. Site is the abbreviation for the sampling site and n refers to number of analyzed specimens per site. AR is the rarefied average allelic richness over loci, and # alleles is the number of alleles found for each locus. H_O_ is the observed and H_E_ the expected heterozygosity, and bold values indicate significant deviations from HWE. (PDF 44 kb) Additional file 6: Pairwise *CO1 F*_ST_ values between all sampling sites. Sampling sites are indicated by the site abbreviations, and letters from A–G indicate GENELAND groups. Colors indicate within-group *F*_ST_ values. Asterisks at site names indicate populations where microsatellite loci were also analyzed. Red and bold *F*_ST_ values indicate significant values. Significance levels were adjusted according to the FDR. (PDF 67 kb) Additional file 7: Pairwise microsatellite *F*_ST_ values between all sampling sites. Sampling sites are indicated by the site abbreviations, and letters from A–G indicate GENELAND groups. Colors represent within-group *F*_ST_ values. Red and bold *F*_ST_ values indicate significant values. Significance levels were adjusted according to the FDR. Below the diagonal, uncorrected *F*_ST_ values are shown; above the diagonal, ENA-corrected *F*_ST_ values are shown. (PDF 42 kb) Additional file 8: Percentage of populations predicted to belong to different GENELAND groups according to the discriminant analyses. (PDF 35 kb)
