# Supplementary material for: Widely distributed and regionally isolated! Drivers of genetic structure in Gammarus fossarum in a human-impacted landscape
Source: BMC Evol Biol. 2016 Jul 29;16:153. doi: 10.1186/s12862-016-0723-z (PMC4966747; doi:10.1186/s12862-016-0723-z)
Supplement: Additional file 1: — Ecological parameters for individual sampling sites. Group indicates the GENELAND group association of the sampling sites. Site is the abbreviation for each sampling site, and geographic coordinates are given in the Gauss–Krueger coordinate system. NA: no data were available for this site. (PDF 66 kb) [file 12862_2016_723_MOESM1_ESM.pdf]

Additional file 1. Ecological parameters for the sampling sites. Group indicates the GENELAND group association of the sampling sites. Site is the abbreviation for the sampling sites and geographic coordinates are given in the Gauss-Krüger coordinate system. NA means that no data were available for this site.

group	site	longitude [GK3]	latitude [GK3]	sub-catchment [no.]	altitude [m]	distance to spring [m]	nature reserve? [1 = yes, 0 = no]	urban area [%]	broadleaf forest [%]	mixed forest [%]	conifer forest [%]	waters [%]	farmland [%]	grassland [%]	channel pattern (MP1) [class]	longitudinal profile (MP2) [class]	water bed structure (MP3) [class]	ecological status [class]	calcium [mg/l]	iron [µg/l]	oxygen [mg/l]	chloride [mg/l]	ammonium [mg/l]	total organic carbon (TOC) [mg/l]	total nitrogen [mg/l]	pH
A	**AA**	3467971	5696423	Möhne	423	3444	1	15.4	2.1	0.0	1.4	0.0	13.1	68.0	5	4	4	NA	78.9	199	11.1	13.6	0.07	1.2	4.3	8.3
	**AL**	3473840	5701645	Lippe	365	0	1	NA	NA	NA	NA	NA	NA	NA	2	2	1	4	NA	NA	NA	NA	NA	NA	NA	NA
	**QB12**	3461411	5681944	Ruhr	474	5626	1	0.0	0.0	0.0	3.1	0.0	0.0	96.9	3	3	2	5	NA	NA	NA	NA	0.05	NA	NA	8.3
	**E11**	3438640	5672688	Lenne	382	1486	0	0.0	0.0	0.0	16.8	0.0	0.0	83.2	2	1	1	3	NA	NA	NA	NA	0.09	NA	NA	7.7
	**E06**	3434942	5674699	Lenne	334	4874	0	21.2	8.3	0.0	0.0	0.0	0.0	70.5	6	5	4	4	47.7	92	11.2	11.8	NA	1.4	3.1	NA
	**E02**	3453327	5672099	Lenne	447	3973	0	24.3	0.9	3.6	0.0	0.0	0.0	71.2	5	4	5	5	37.1	147	10.3	14.6	0.03	2.4	2.8	7.8
	**GS**	3441171	5701881	Möhne	270	11525	1	0.0	0.0	0.0	65.0	0.0	0.0	35.0	2	1	1	2	8.5	1100	10.6	6.5	NA	6.2	1.2	NA
	**QB24**	3442318	5677813	Ruhr	347	4706	0	0.0	0.0	0.0	0.0	0.0	0.0	100.0	NA	NA	NA	NA	45.1	111	10.5	12.8	0.03	1.6	3.7	7.6
	**QB10**	3453286	5701356	Möhne	314	8716	0	0.0	0.0	0.0	0.0	0.0	0.0	100.0	NA	NA	NA	NA	58.6	197	10.5	24.8	0.09	3.3	3.7	7.7
	**QB17**	3445686	5678146	Ruhr	342	6845	0	0.0	7.1	24.7	0.0	0.0	0.0	68.2	2	1	1	2	43.8	48	10.3	9.0	0.07	1.3	4.1	8.3
	**QB23**	3447495	5677478	Ruhr	369	4550	0	13.6	13.0	0.0	0.0	0.0	0.0	73.4	4	7	3	2	30.7	76	9.8	11.8	0.08	1.4	3.4	7.9
	**VR7**	3445543	5686406	Ruhr	399	2172	0	0.0	0.0	0.0	68.8	1.2	0.0	29.9	3	3	2	4	68.0	172	10.8	10.6	0.08	1.4	5.9	7.4
	**QB22**	3453172	5688126	Ruhr	312	13895	0	9.4	22.8	0.0	0.6	0.0	27.9	39.4	3	5	4	2	43.4	98	10.8	12.1	0.04	1.3	4.1	7.9
	**LE**	3452684	5673900	Ruhr	502	785	0	0.0	14.2	6.6	49.8	0.0	0.0	29.4	5	4	2	2	20.3	67	10.2	8.1	0.09	1.0	2.2	7.9
	**LO**	3458556	5702557	Möhne	309	11034	1	0.0	4.2	0.0	0.0	0.0	0.0	95.8	3	3	3	2	74.6	63	10.1	35.3	NA	1.7	3.4	NA
	**ME**	3466980	5687670	Ruhr	442	5723	0	98.8	0.0	0.0	0.5	0.0	0.0	0.7	6	5	6	4	32.3	40	10.5	8.2	0.08	1.2	2.5	7.8
	**KL6**	3465023	5697740	Möhne	451	1420	1	11.4	0.0	3.0	0.0	0.0	0.0	85.6	NA	NA	NA	NA	NA	NA	NA	NA	0.08	NA	NA	7.8
	**VR17**	3457758	5678654	Ruhr	509	3363	0	12.0	13.0	8.7	13.0	0.0	0.0	53.3	7	7	7	2	24.5	62	10.8	8.7	0.06	1.2	2.6	8.1
	**RO1**	3434318	5679606	Ruhr	527	1500	0	19.8	0.0	0.0	34.2	0.4	0.0	45.6	2	7	3	5	NA	NA	NA	NA	NA	NA	NA	NA
	**RO2**	3431365	5686987	Ruhr	272	11754	0	4.9	0.8	24.1	33.1	0.0	0.0	37.0	5	5	5	5	23.6	107	10.2	6.6	0.12	1.3	3.0	7.9
	**KL9**	3434351	5682378	Ruhr	401	5187	0	45.6	37.1	0.0	0.0	0.0	0.2	17.1	3	5	4	5	23.6	107	10.2	6.6	0.07	1.3	3.0	7.8
	**SB**	3460026	5694422	Ruhr	346	3615	0	4.2	0.0	5.9	13.7	0.3	0.0	75.8	2	3	2	3	23.8	187	10.5	8.5	0.08	2.4	2.2	8.2
	**QB27**	3446183	5688992	Ruhr	295	5697	0	14.2	16.4	0.0	0.0	0.0	0.0	69.5	NA	NA	NA	NA	68.0	78	10.8	10.6	0.07	1.4	5.9	8.1
	**KL3**	3432816	5681104	Ruhr	403	3219	0	1.4	14.2	0.0	29.0	1.2	0.0	54.2	5	5	5	2	16.2	80	10.6	4.8	0.08	1.3	2.3	8.0
B	**BB**	3466645	5668829	Eder	524	2506	0	0.0	16.9	0.2	11.5	0.0	0.0	71.3	3	4	2	2	16.9	90	10.4	5.9	0.08	1.6	1.3	7.6
	**HSK10**	3458921	5685865	Ruhr	433	1435	0	0.1	40.1	40.1	9.8	0.0	0.0	9.9	NA	NA	NA	NA	30.9	93	10.8	10.2	0.08	1.5	2.6	7.4
	**E04**	3409316	5681433	Lenne	305	2163	0	0.0	55.1	3.2	29.8	0.0	0.0	11.9	NA	NA	NA	NA	NA	NA	NA	NA	0.05	NA	NA	8.2
	**NL**	3464929	5675392	Ruhr	611	4134	1	0.0	2.9	0.0	4.5	0.0	0.0	92.6	3	4	2	3	20.4	554	10.8	17.4	0.11	2.4	1.2	8.1
	**HSK6b**	3455374	5686738	Ruhr	399	4394	0	24.1	16.1	0.0	6.5	0.0	0.0	53.3	4	3	2	4	NA	NA	NA	NA	NA	NA	NA	NA
	**PL1**	3457450	5680116	Ruhr	466	5145	0	53.6	1.5	0.0	8.3	0.0	1.0	35.7	3	1	1	2	24.5	62	10.8	8.7	NA	1.2	2.6	NA
	**PL2**	3458169	5676718	Ruhr	629	1156	1	0.0	30.3	6.7	16.2	0.0	0.0	46.8	3	4	3	2	24.5	62	10.8	8.7	0.08	1.2	2.6	7.8
	**NG**	3462376	5675757	Ruhr	574	4783	1	0.0	57.6	0.0	35.8	0.0	0.0	6.6	NA	NA	NA	NA	17.0	53	11.1	9.0	NA	1.4	1.2	NA
	**KL13**	3410745	5675226	Lenne	307	5840	0	16.5	23.0	5.0	1.9	0.0	15.3	38.2	4	3	4	3	23.1	155	10.7	17.1	NA	2.0	2.5	NA
	**SO**	3458196	5673862	Lenne	537	2980	1	26.8	0.0	0.0	15.5	0.0	0.0	57.6	3	3	5	2	NA	NA	NA	NA	0.04	NA	NA	7.5
	**VR2**	3460431	5673996	Lenne	654	385	1	0.0	77.6	1.2	21.2	0.0	0.0	0.0	2	3	2	2	NA	NA	NA	NA	0.09	NA	NA	7.6
	**VA1**	3459155	5678350	Ruhr	593	3040	0	0.0	0.8	0.0	45.5	0.0	0.0	53.7	3	5	3	2	NA	NA	NA	NA	0.08	NA	NA	7.8
	**VR5**	3458707	5683775	Ruhr	411	9494	0	19.1	8.5	0.0	36.9	0.0	0.0	35.5	5	6	4	2	26.7	44	11.1	7.6	0.08	1.6	2.8	7.8
C	**HB1**	3478675	5677590	Eder	458	7277	0	0.0	18.9	0.0	23.2	1.4	0.0	56.5	2	2	1	2	28.4	87	10.5	14.7	0.08	1.9	1.4	7.8
	**HB2**	3474569	5675459	Eder	554	1850	1	0.0	4.4	31.9	55.1	0.0	0.0	8.6	4	5	2	2	NA	NA	NA	NA	0.07	NA	NA	7.7
	**ND**	3478218	5682426	Eder	564	2076	0	NA	NA	NA	NA	NA	NA	NA	NA	NA	NA	NA	NA	NA	NA	NA	0.08	NA	NA	7.8
	**NH**	3467985	5671089	Eder	540	3751	1	8.4	0.0	18.7	0.0	0.0	0.0	72.9	4	4	2	2	18.7	82	10.6	6.5	NA	1.6	1.5	NA
	**RU3**	3466754	5681626	Ruhr	504	8222	1	40.1	11.0	0.0	6.2	0.0	0.0	42.7	6	6	4	3	18.4	181	10.8	11.6	0.07	2.1	1.8	7.8
	**RU4**	3467531	5676847	Ruhr	622	2614	1	0.0	3.1	0.0	41.0	0.0	0.0	55.9	4	5	2	3	18.4	181	10.8	11.6	0.07	2.1	1.8	7.8
D	**VR16**	3404615	5697289	Ruhr	184	2789	1	0.0	6.2	0.0	39.0	0.0	0.0	54.8	3	4	3	3	28.4	469	10.8	22.3	0.07	4.2	2.9	7.9
	**VR12**	3395008	5671675	Volme	391	1919	1	0.0	43.4	1.2	37.7	0.0	0.0	17.7	2	3	2	3	24.0	140	9.9	17.0	0.07	1.6	3.0	7.9
	**VR23**	3393785	5683027	Volme	291	2012	0	1.1	0.0	0.5	0.3	0.0	0.0	98.1	7	7	7	4	23.4	115	11.1	33.1	0.10	2.3	4.3	8.0
	**GB**	3408047	5691018	Lenne	268	5884	0	3.0	1.2	0.0	38.6	0.0	0.0	57.2	6	4	4	2	31.5	64	10.7	18.8	0.08	1.9	3.6	8.2
	**NB**	3400329	5688143	Lenne	224	3502	0	6.5	23.6	9.5	28.9	0.0	0.0	31.4	NA	NA	NA	NA	19.9	121	10.6	18.3	0.03	1.9	3.5	7.7
	**VR11**	3406403	5698078	Ruhr	184	3511	0	0.0	59.4	0.0	0.0	0.0	0.0	40.6	1	1	1	4	43.9	244	10.2	24.9	NA	3.9	3.4	NA
E	**KL14**	3426167	5696529	Ruhr	294	2215	1	5.3	10.8	0.0	8.0	2.6	7.7	65.6	4	3	3	2	76.0	218	11.1	18.0	NA	1.4	4.9	NA
	**QB29**	3425599	5681574	Ruhr	402	2682	0	0.0	3.8	0.0	69.7	0.0	0.0	26.5	NA	NA	NA	NA	42.1	186	10.7	11.9	NA	1.9	3.9	NA
	**E01**	3410771	5681557	Lenne	237	1843	0	0.0	12.8	0.0	68.5	0.0	0.0	18.8	NA	NA	NA	NA	NA	NA	NA	NA	0.08	NA	NA	7.9
F	**KL2**	3417114	5663535	Lenne	347	1861	0	16.5	0.0	0.2	0.0	0.0	0.0	83.3	NA	NA	NA	NA	21.5	290	9.9	41.8	0.07	3.7	3.8	7.6
G	**KL15**	3409854	5660640	Lenne	416	3276	0	0.0	2.0	0.0	0.0	0.0	0.0	98.0	2	3	1	3	19.4	1055	10.5	18.5	0.29	3.5	3.4	7.6
