# Supplementary material for: Widely distributed and regionally isolated! Drivers of genetic structure in Gammarus fossarum in a human-impacted landscape
Source: BMC Evol Biol. 2016 Jul 29;16:153. doi: 10.1186/s12862-016-0723-z (PMC4966747; doi:10.1186/s12862-016-0723-z)
Supplement: Additional file 2: — DNA salt-extraction protocol, modified from Sunnucks & Hales [44]. (PDF 51 kb) [file 12862_2016_723_MOESM2_ESM.pdf]

Additional file 2. DNA salt-extraction protocol; modified from Sunnucks & Hales [44].

- Incubate a small piece of tissue in 600 µl of TNES buffer (50 mM Tris, pH 7.5, 400 mM NaCl, 20 mM EDTA, 0.5% SOS) with 10 - 20 µl of Proteinase K (10 mg/ml) for 3 h at 55°C or 37°C overnight.
- Add 170 µl of 5 M NaCl and mix for 15 s to precipitate proteins.
- Centrifuge for 5 min at 18,400 rcf, pipette the clear supernatant into a new tube, and repeat the centrifugation for 5 min. Pipette the clear supernatant into a new tube and discard the protein pellet.
- Add 800 µl of ice-cold 100% ethanol, invert tubes multiple times, and centrifuge for 15 min at 18,400 rcf in a cooling centrifuge to pellet the DNA.
- Discard the clear supernatant (do not discard the DNA pellet), add 180 µl of 70% ethanol, and centrifuge again for 15 min at 18,400 rcf in a cooling centrifuge.
- Remove the supernatant and air dry the pellet.
- Dissolve the DNA in 50 µl of TE-minimum buffer or sterile water.
