# Supplementary material for: Widely distributed and regionally isolated! Drivers of genetic structure in Gammarus fossarum in a human-impacted landscape
Source: BMC Evol Biol. 2016 Jul 29;16:153. doi: 10.1186/s12862-016-0723-z (PMC4966747; doi:10.1186/s12862-016-0723-z)
Supplement: Additional file 3: — CO1 haplotype information for each sampling site. Group indicates the GENELAND group association of the sampling sites, site is the abbreviation for the sampling site, and n refers to number of analyzed specimens per site. # H gives the total number of haplotypes at a site and private H is the number of private haplotypes. H diversity is the haplotype diversity and H1 to H40 represent the different haplotypes. (PDF 67 kb) [file 12862_2016_723_MOESM3_ESM.pdf]

Additional file 3. CO1 haplotype information for each sampling site. Group indicates the GENELAND group association of the sampling sites, site is the abbreviation for the sampling site, and n indicates the number of analyzed specimens per site. # H gives the total number of haplotypes at a site, and private H is the number of private haplotypes. H diversity is the haplotype diversity, π the nucleotide diversity and H1 to H40 represent the different haplotypes.

group	site	n	# H	private H	H diversity	π	H1	H2	H3	H4	H5	H6	H7	H8	H9	H10	H11	H12	H13	H14	H15	H16	H17	H18	H19	H20	H21	H22	H23	H24	H25	H26	H27	H28	H29	H30	H31	H32	H33	H34	H35	H36	H37	H38	H39	H40
A	**AA**	17	3	0	0.404	0.130	13	0	0	0	0	0	0	0	0	0	0	0	0	0	0	0	0	0	0	0	3	1	0	0	0	0	0	0	0	0	0	0	0	0	0	0	0	0	0	0
	**AL**	30	1	0	0.000	0.000	30	0	0	0	0	0	0	0	0	0	0	0	0	0	0	0	0	0	0	0	0	0	0	0	0	0	0	0	0	0	0	0	0	0	0	0	0	0	0	0
	**QB12**	36	2	0	0.157	0.077	33	0	0	0	0	0	0	0	0	0	0	0	0	0	0	0	0	0	3	0	0	0	0	0	0	0	0	0	0	0	0	0	0	0	0	0	0	0	0	0
	**E11**	22	2	0	0.091	0.081	21	0	0	0	0	0	0	0	0	0	0	0	0	0	0	0	0	0	1	0	0	0	0	0	0	0	0	0	0	0	0	0	0	0	0	0	0	0	0	0
	**E06**	15	2	1	0.248	0.131	13	0	0	0	0	0	0	0	0	0	0	0	0	0	0	0	2	0	0	0	0	0	0	0	0	0	0	0	0	0	0	0	0	0	0	0	0	0	0	0
	**E02**	54	2	0	0.037	0.035	53	0	0	0	0	0	0	0	0	0	0	0	0	0	0	0	0	0	1	0	0	0	0	0	0	0	0	0	0	0	0	0	0	0	0	0	0	0	0	0
	**GS**	30	1	0	0.000	0.000	30	0	0	0	0	0	0	0	0	0	0	0	0	0	0	0	0	0	0	0	0	0	0	0	0	0	0	0	0	0	0	0	0	0	0	0	0	0	0	0
	**QB24**	48	1	0	0.000	0.000	48	0	0	0	0	0	0	0	0	0	0	0	0	0	0	0	0	0	0	0	0	0	0	0	0	0	0	0	0	0	0	0	0	0	0	0	0	0	0	0
	**QB10**	27	3	1	0.145	0.090	25	0	0	1	0	0	0	0	0	0	0	0	0	0	0	0	0	0	1	0	0	0	0	0	0	0	0	0	0	0	0	0	0	0	0	0	0	0	0	0
	**QB17**	36	1	0	0.000	0.000	36	0	0	0	0	0	0	0	0	0	0	0	0	0	0	0	0	0	0	0	0	0	0	0	0	0	0	0	0	0	0	0	0	0	0	0	0	0	0	0
	**QB23**	36	1	0	0.000	0.000	36	0	0	0	0	0	0	0	0	0	0	0	0	0	0	0	0	0	0	0	0	0	0	0	0	0	0	0	0	0	0	0	0	0	0	0	0	0	0	0
	**VR7**	48	1	0	0.000	0.000	48	0	0	0	0	0	0	0	0	0	0	0	0	0	0	0	0	0	0	0	0	0	0	0	0	0	0	0	0	0	0	0	0	0	0	0	0	0	0	0
	**QB22**	58	1	0	0.000	0.000	58	0	0	0	0	0	0	0	0	0	0	0	0	0	0	0	0	0	0	0	0	0	0	0	0	0	0	0	0	0	0	0	0	0	0	0	0	0	0	0
	**LE**	53	3	1	0.177	0.068	48	0	0	0	1	0	0	0	0	0	0	0	0	0	0	0	0	0	4	0	0	0	0	0	0	0	0	0	0	0	0	0	0	0	0	0	0	0	0	0
	**LO**	30	1	0	0.000	0.000	30	0	0	0	0	0	0	0	0	0	0	0	0	0	0	0	0	0	0	0	0	0	0	0	0	0	0	0	0	0	0	0	0	0	0	0	0	0	0	0
	**ME**	18	2	0	0.111	0.096	17	0	0	0	0	0	0	0	0	0	0	0	0	0	0	0	0	0	0	0	1	0	0	0	0	0	0	0	0	0	0	0	0	0	0	0	0	0	0	0
	**KL6**	60	1	0	0.000	0.000	60	0	0	0	0	0	0	0	0	0	0	0	0	0	0	0	0	0	0	0	0	0	0	0	0	0	0	0	0	0	0	0	0	0	0	0	0	0	0	0
	**VR17**	44	3	1	0.394	0.072	33	0	1	0	0	0	0	0	0	0	0	0	0	0	0	0	0	0	10	0	0	0	0	0	0	0	0	0	0	0	0	0	0	0	0	0	0	0	0	0
	**RO1**	20	1	0	0.000	0.000	20	0	0	0	0	0	0	0	0	0	0	0	0	0	0	0	0	0	0	0	0	0	0	0	0	0	0	0	0	0	0	0	0	0	0	0	0	0	0	0
	**RO2**	25	2	0	0.153	0.092	23	0	0	0	0	0	2	0	0	0	0	0	0	0	0	0	0	0	0	0	0	0	0	0	0	0	0	0	0	0	0	0	0	0	0	0	0	0	0	0
	**KL9**	59	1	0	0.000	0.000	59	0	0	0	0	0	0	0	0	0	0	0	0	0	0	0	0	0	0	0	0	0	0	0	0	0	0	0	0	0	0	0	0	0	0	0	0	0	0	0
	**SB**	58	3	2	0.101	0.054	55	1	0	0	0	0	0	0	0	0	0	0	0	0	0	0	0	2	0	0	0	0	0	0	0	0	0	0	0	0	0	0	0	0	0	0	0	0	0	0
	**QB27**	58	1	0	0.000	0.000	58	0	0	0	0	0	0	0	0	0	0	0	0	0	0	0	0	0	0	0	0	0	0	0	0	0	0	0	0	0	0	0	0	0	0	0	0	0	0	0
	**KL3**	71	4	2	0.385	0.068	55	0	0	0	0	0	8	0	0	2	6	0	0	0	0	0	0	0	0	0	0	0	0	0	0	0	0	0	0	0	0	0	0	0	0	0	0	0	0	0
B	**BB**	29	1	0	0.000	0.000	0	0	0	0	0	0	0	0	0	0	0	0	0	0	0	0	0	0	29	0	0	0	0	0	0	0	0	0	0	0	0	0	0	0	0	0	0	0	0	0
	**HSK10**	12	1	0	0.000	0.000	0	0	0	0	0	0	0	0	0	0	0	0	0	0	0	0	0	0	12	0	0	0	0	0	0	0	0	0	0	0	0	0	0	0	0	0	0	0	0	0
	**E04**	29	1	0	0.000	0.000	0	0	0	0	0	0	0	0	0	0	0	0	0	0	0	0	0	0	29	0	0	0	0	0	0	0	0	0	0	0	0	0	0	0	0	0	0	0	0	0
	**NL**	12	2	0	0.167	0.134	1	0	0	0	0	0	0	0	0	0	0	0	0	0	0	0	0	0	11	0	0	0	0	0	0	0	0	0	0	0	0	0	0	0	0	0	0	0	0	0
	**HSK6b**	26	2	0	0.443	0.074	8	0	0	0	0	0	0	0	0	0	0	0	0	0	0	0	0	0	18	0	0	0	0	0	0	0	0	0	0	0	0	0	0	0	0	0	0	0	0	0
	**PL1**	25	2	0	0.520	0.030	13	0	0	0	0	0	0	0	0	0	0	0	0	0	0	0	0	0	12	0	0	0	0	0	0	0	0	0	0	0	0	0	0	0	0	0	0	0	0	0
	**PL2**	82	2	1	0.024	0.024	0	0	0	0	0	0	0	0	0	0	0	0	0	0	0	0	0	0	81	0	0	0	0	0	0	0	1	0	0	0	0	0	0	0	0	0	0	0	0	0
	**NG**	46	3	1	0.478	0.052	15	0	0	0	0	0	0	0	0	0	0	0	0	0	0	0	0	0	0	0	0	0	0	0	0	0	0	0	0	0	30	1	0	0	0	0	0	0	0	0
	**KL13**	68	5	1	0.677	0.025	21	0	0	0	0	0	0	0	0	0	0	0	0	0	0	0	0	0	16	1	0	0	0	0	0	1	0	0	0	29	0	0	0	0	0	0	0	0	0	0
	**SO**	14	2	0	0.363	0.130	3	0	0	0	0	0	0	0	0	0	0	0	0	0	0	0	0	0	11	0	0	0	0	0	0	0	0	0	0	0	0	0	0	0	0	0	0	0	0	0
	**VR2**	56	4	3	0.138	0.062	0	0	0	0	0	0	0	0	0	0	0	0	0	0	0	0	0	0	52	0	0	0	2	1	1	0	0	0	0	0	0	0	0	0	0	0	0	0	0	0
	**VA1**	20	3	0	0.279	0.124	2	0	0	0	0	0	0	0	0	0	0	0	0	0	0	0	0	0	17	0	0	0	0	0	0	0	0	0	0	0	1	0	0	0	0	0	0	0	0	0
	**VR5**	48	2	0	0.311	0.071	9	0	0	0	0	0	0	0	0	0	0	0	0	0	0	0	0	0	39	0	0	0	0	0	0	0	0	0	0	0	0	0	0	0	0	0	0	0	0	0
C	**HB1**	16	2	0	0.125	0.106	0	0	0	0	0	0	0	0	0	0	0	0	0	0	0	0	0	0	1	0	15	0	0	0	0	0	0	0	0	0	0	0	0	0	0	0	0	0	0	0
	**HB2**	20	1	0	0.000	0.000	0	0	0	0	0	0	0	0	0	0	0	0	0	0	0	0	0	0	0	0	20	0	0	0	0	0	0	0	0	0	0	0	0	0	0	0	0	0	0	0
	**ND**	24	3	0	0.540	0.062	9	0	0	0	0	0	0	0	0	0	0	0	0	0	0	0	0	0	0	0	14	1	0	0	0	0	0	0	0	0	0	0	0	0	0	0	0	0	0	0
	**NH**	58	4	0	0.304	0.073	2	0	0	0	0	0	0	0	0	0	0	0	0	0	0	0	0	0	0	1	48	0	0	0	0	0	0	0	0	0	7	0	0	0	0	0	0	0	0	0
	**RU3**	52	2	0	0.462	0.043	18	0	0	0	0	0	0	0	0	0	0	0	0	0	0	0	0	0	0	0	34	0	0	0	0	0	0	0	0	0	0	0	0	0	0	0	0	0	0	0
	**RU4**	7	1	0	0.000	0.000	0	0	0	0	0	0	0	0	0	0	0	0	0	0	0	0	0	0	0	0	7	0	0	0	0	0	0	0	0	0	0	0	0	0	0	0	0	0	0	0
D	**VR16**	36	3	2	0.110	0.070	0	0	0	0	0	0	0	0	0	0	0	0	0	0	0	0	0	0	0	0	0	0	0	0	0	0	0	0	0	0	0	0	34	0	0	0	1	1	0	0
	**VR12**	48	3	2	0.122	0.063	0	0	0	0	0	0	0	0	0	0	0	0	0	0	0	0	0	0	0	0	0	0	0	0	0	0	0	0	0	0	0	0	45	0	0	1	0	0	0	2
	**VR23**	56	2	1	0.103	0.054	0	0	0	0	0	0	0	0	0	0	0	0	0	0	0	0	0	0	0	0	0	0	0	0	0	0	0	0	0	0	0	0	53	0	0	0	0	0	3	0
	**GB**	29	2	1	0.069	0.063	0	0	0	0	0	0	0	0	0	0	0	0	0	0	0	0	0	0	0	0	0	0	0	0	0	0	0	0	0	0	0	0	28	0	1	0	0	0	0	0
	**NB**	37	1	0	0.000	0.000	0	0	0	0	0	0	0	0	0	0	0	0	0	0	0	0	0	0	0	0	0	0	0	0	0	0	0	0	0	0	0	0	37	0	0	0	0	0	0	0
	**VR11**	47	2	1	0.043	0.040	0	0	0	0	0	0	0	0	0	0	0	0	0	0	0	0	0	0	0	0	0	0	0	0	0	0	0	0	0	0	0	0	46	1	0	0	0	0	0	0
E	**KL14**	60	6	4	0.369	0.072	9	0	0	0	0	0	0	0	0	0	0	47	1	1	1	1	0	0	0	0	0	0	0	0	0	0	0	0	0	0	0	0	0	0	0	0	0	0	0	0
	**QB29**	46	4	2	0.312	0.085	3	0	0	0	0	4	38	0	1	0	0	0	0	0	0	0	0	0	0	0	0	0	0	0	0	0	0	0	0	0	0	0	0	0	0	0	0	0	0	0
	**E01**	14	2	1	0.264	0.136	2	0	0	0	0	0	0	12	0	0	0	0	0	0	0	0	0	0	0	0	0	0	0	0	0	0	0	0	0	0	0	0	0	0	0	0	0	0	0	0
F	**KL2**	57	1	1	0.000	0.000	0	0	0	0	0	0	0	0	0	0	0	0	0	0	0	0	0	0	0	0	0	0	0	0	0	0	0	57	0	0	0	0	0	0	0	0	0	0	0	0
G	**KL15**	59	1	1	0.000	0.000	0	0	0	0	0	0	0	0	0	0	0	0	0	0	0	0	0	0	0	0	0	0	0	0	0	0	0	0	59	0	0	0	0	0	0	0	0	0	0	0
	**Total**	2086		30			1017	1	1	1	1	4	48	12	1	2	6	47	1	1	1	1	2	2	348	2	142	2	2	1	1	1	1	57	59	29	38	1	243	1	1	1	1	1	3	2
