# Supplementary material for: Widely distributed and regionally isolated! Drivers of genetic structure in Gammarus fossarum in a human-impacted landscape
Source: BMC Evol Biol. 2016 Jul 29;16:153. doi: 10.1186/s12862-016-0723-z (PMC4966747; doi:10.1186/s12862-016-0723-z)
Supplement: Additional file 4: — a: Sampling locations of specimens used in the phylogeny. Symbols in the map correspond with symbols in b to indicate sampling locations of used sequences; the red square indicates the Sauerland area in which the specimens belonging to the main haplotypes were sampled. b: Neighbor-joining tree of main haplotypes and additional sequences belonging to clades 3, 10, 11, 12 and 13 (sensu Weiss et al. [10]). The tree is drawn to scale, with branch lengths (next to the branches) in the same units as those of the evolutionary distances used to infer the phylogenetic tree (K2P method). (PDF 2114 kb) [file 12862_2016_723_MOESM4_ESM.pdf]

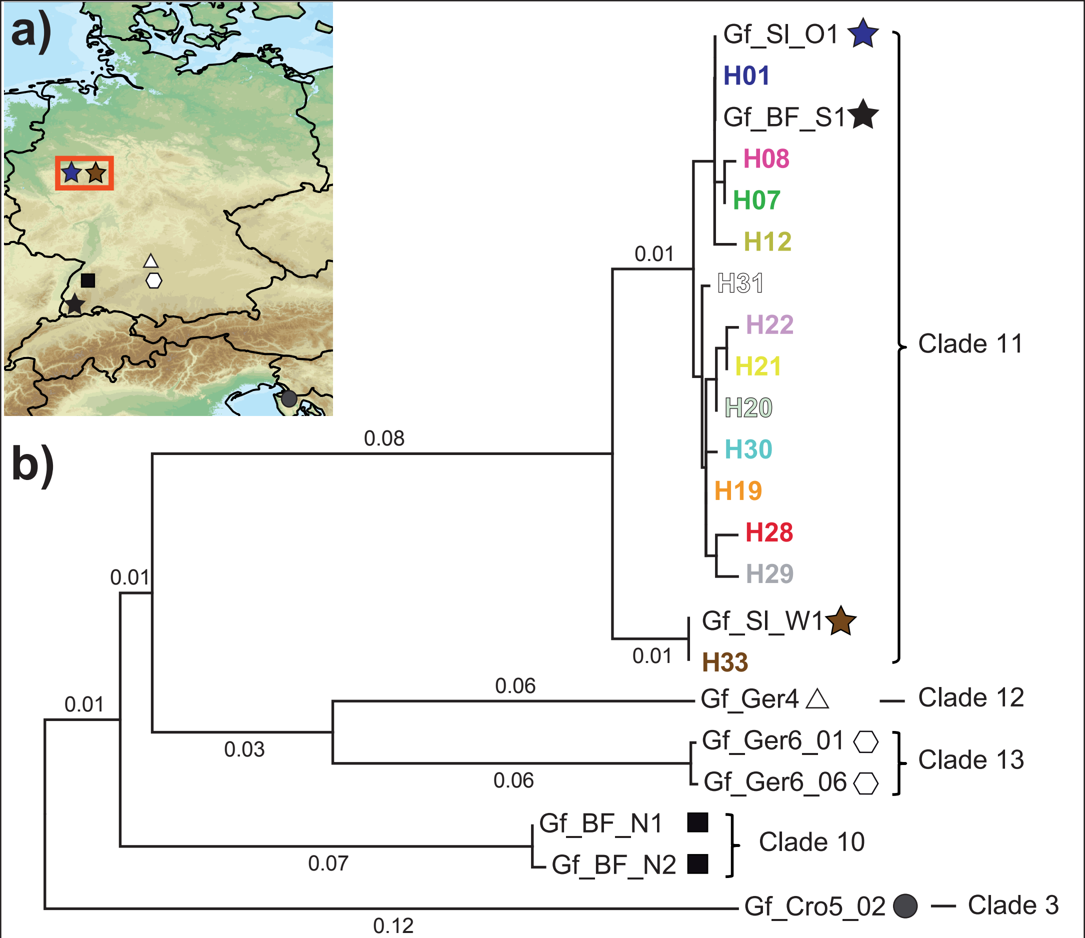

Additional file 4 a: Sampling locations of specimens used in the phylogeny. Symbols in the map correspond with symbols in **b** to indicate sampling locations of used sequences; the red square indicates the Sauerland area in which the specimens belonging to the main haplotypes were sampled. **b:** Neighbor-joining tree of main haplotypes and additional sequences belonging to clades 3, 10, 11, 12 and 13 (sensu Weiss et al. [10]). The tree is drawn to scale, with branch lengths (next to the branches) in the same units as those of the evolutionary distances used to infer the phylogenetic tree (K2P method).
