# Supplementary material for: Widely distributed and regionally isolated! Drivers of genetic structure in Gammarus fossarum in a human-impacted landscape
Source: BMC Evol Biol. 2016 Jul 29;16:153. doi: 10.1186/s12862-016-0723-z (PMC4966747; doi:10.1186/s12862-016-0723-z)
Supplement: Additional file 5: — Microsatellite loci information for the different sampling sites. Site is the abbreviation for the sampling site and n refers to number of analyzed specimens per site. AR is the rarefied average allelic richness over loci, and # alleles is the number of alleles found for each locus. HO is the observed and HE the expected heterozygosity, and bold values indicate significant deviations from HWE. (PDF 44 kb) [file 12862_2016_723_MOESM5_ESM.pdf]

**Additional file 5:** Microsatellite loci information for the different sampling sites. Site is the abbreviation for the sampling site and n refers to number of analyzed specimens per site. AR is the rarefied average allelic richness over loci and # alleles the number of alleles found for each locus. $H_O$ is the observed and $H_E$ the expected heterozygosity and bold values indicate significant deviations from HWE.

| site | n | AR | locus | # alleles | private alleles | $H_O$ | $H_E$ | null allele frequency |
|---|---|---|---|---|---|---|---|---|
| **AL** | 30 | 5.33 | Gamfos28 | 16 | 4 | **0.72** | 0.90 | 0.08 |
| | | | Gamfos13 | 2 | 0 | 0.07 | 0.07 | 0.00 |
| | | | Gamfos18 | 8 | 0 | 0.37 | 0.36 | 0.00 |
| | | | Gamfos10 | 2 | 0 | **0.07** | 0.36 | 0.23 |
| **E06** | 15 | 2.50 | Gamfos28 | 3 | 0 | **0.27** | 0.40 | 0.10 |
| | | | Gamfos13 | 1 | 0 | nA | nA | 0.00 |
| | | | Gamfos18 | 4 | 1 | 0.27 | 0.25 | 0.00 |
| | | | Gamfos10 | 2 | 0 | 0.13 | 0.13 | 0.00 |
| **E02** | 54 | 3.86 | Gamfos28 | 6 | 0 | **0.13** | 0.57 | 0.29 |
| | | | Gamfos13 | 2 | 0 | 0.30 | 0.39 | 0.07 |
| | | | Gamfos18 | 8 | 0 | 0.69 | 0.72 | 0.00 |
| | | | Gamfos10 | 4 | 0 | 0.31 | 0.29 | 0.00 |
| **LE** | 53 | 2.50 | Gamfos28 | 3 | 0 | **0.08** | 0.31 | 0.20 |
| | | | Gamfos13 | 3 | 1 | **0.68** | 0.52 | 0.00 |
| | | | Gamfos18 | 5 | 0 | 0.26 | 0.24 | 0.00 |
| | | | Gamfos10 | 2 | 1 | 0.02 | 0.02 | 0.00 |
| **SB** | 58 | 2.70 | Gamfos28 | 4 | 0 | 0.60 | 0.68 | 0.04 |
| | | | Gamfos13 | 2 | 0 | 0.02 | 0.02 | 0.00 |
| | | | Gamfos18 | 4 | 0 | 0.22 | 0.22 | 0.00 |
| | | | Gamfos10 | 4 | 0 | **0.12** | 0.24 | 0.14 |
| **PL1** | 25 | 3.84 | Gamfos28 | 3 | 0 | **0.16** | 0.54 | 0.25 |
| | | | Gamfos13 | 2 | 0 | **0.24** | 0.49 | 0.16 |
| | | | Gamfos18 | 9 | 0 | **0.56** | 0.67 | 0.06 |
| | | | Gamfos10 | 4 | 1 | 0.24 | 0.37 | 0.10 |
| **NG** | 46 | 4.62 | Gamfos28 | 10 | 1 | 0.77 | 0.88 | 0.05 |
| | | | Gamfos13 | 1 | 0 | nA | nA | 0.00 |
| | | | Gamfos18 | 9 | 0 | 0.65 | 0.65 | 0.00 |
| | | | Gamfos10 | 3 | 0 | **0.17** | 0.30 | 0.13 |
| **NH** | 58 | 3.86 | Gamfos28 | 8 | 1 | **0.27** | 0.59 | 0.18 |
| | | | Gamfos13 | 2 | 1 | 0.03 | 0.03 | 0.00 |
| | | | Gamfos18 | 10 | 0 | 0.79 | 0.77 | 0.01 |
| | | | Gamfos10 | 3 | 0 | **0.19** | 0.29 | 0.11 |
| **RU3** | 52 | 4.69 | Gamfos28 | 9 | 0 | **0.31** | 0.83 | 0.28 |
| | | | Gamfos13 | 2 | 0 | 0.12 | 0.14 | 0.05 |
| | | | Gamfos18 | 8 | 0 | 0.62 | 0.68 | 0.03 |
| | | | Gamfos10 | 4 | 0 | 0.60 | 0.57 | 0.00 |
| **GB** | 29 | 2.65 | Gamfos28 | 6 | 0 | **0.31** | 0.58 | 0.17 |
| | | | Gamfos13 | 1 | 0 | nA | nA | 0.00 |
| | | | Gamfos18 | 3 | 0 | 0.10 | 0.10 | 0.00 |
| | | | Gamfos10 | 3 | 0 | **0.10** | 0.54 | 0.28 |
