# Supplementary material for: Widely distributed and regionally isolated! Drivers of genetic structure in Gammarus fossarum in a human-impacted landscape
Source: BMC Evol Biol. 2016 Jul 29;16:153. doi: 10.1186/s12862-016-0723-z (PMC4966747; doi:10.1186/s12862-016-0723-z)
Supplement: Additional file 7: — Pairwise microsatellite F ST values between all sampling sites. Sampling sites are indicated by the site abbreviations, and letters from A–G indicate GENELAND groups. Colors represent within-group F ST values. Red and bold F ST values indicate significant values. Significance levels were adjusted according to the FDR. Below the diagonal, uncorrected F ST values are shown; above the diagonal, ENA-corrected F ST values are shown. (PDF 42 kb) [file 12862_2016_723_MOESM7_ESM.pdf]

Additional file 7: Pairwise microsatellite F_{ST} values between all sampling sites. Sampling sites are indicated by the site abbreviations, and letters from A–G indicate GENELAND groups. Colors indicate within-group F_{ST} values. Red and bold F_{ST} values indicate significant values (all). Significance levels were adjusted according to the FDR. Below the diagonal, uncorrected F_{ST} values are shown; above the diagonal, ENA-corrected F_{ST} values are shown.

		A					B		C		D
		AL	E06	E02	LE	SB	PL1	NG	NH	RU3	GB
A	AL		**0.41**	**0.36**	**0.49**	**0.12**	**0.39**	**0.35**	**0.38**	**0.07**	**0.22**
	E06	**0.37**		**0.25**	**0.18**	**0.48**	**0.30**	**0.48**	**0.51**	**0.32**	**0.26**
	E02	**0.32**	**0.24**		**0.27**	**0.46**	**0.20**	**0.34**	**0.32**	**0.27**	**0.32**
	LE	**0.45**	**0.17**	**0.25**		**0.54**	**0.22**	**0.57**	**0.57**	**0.42**	**0.40**
	SB	**0.11**	**0.44**	**0.43**	**0.50**		**0.49**	**0.50**	**0.51**	**0.12**	**0.20**
B	PL1	**0.34**	**0.30**	**0.16**	**0.20**	**0.45**		**0.40**	**0.40**	**0.31**	**0.36**
	NG	**0.32**	**0.47**	**0.32**	**0.54**	**0.47**	**0.36**		**0.17**	**0.28**	**0.46**
C	NH	**0.34**	**0.48**	**0.31**	**0.55**	**0.47**	**0.36**	**0.14**		**0.27**	**0.48**
	RU3	**0.06**	**0.33**	**0.27**	**0.41**	**0.12**	**0.29**	**0.27**	**0.25**		**0.16**
D	GB	**0.16**	**0.26**	**0.31**	**0.37**	**0.16**	**0.34**	**0.42**	**0.43**	**0.16**	
