# Supplementary material for: Widely distributed and regionally isolated! Drivers of genetic structure in Gammarus fossarum in a human-impacted landscape
Source: BMC Evol Biol. 2016 Jul 29;16:153. doi: 10.1186/s12862-016-0723-z (PMC4966747; doi:10.1186/s12862-016-0723-z)
Supplement: Additional file 8: — Percentage of populations predicted to belong to different GENELAND groups according to the discriminant analyses. (PDF 35 kb) [file 12862_2016_723_MOESM8_ESM.pdf]

Additional file 8: Percentage of populations, predicted to belong to the different GENELAND groups according to the discriminant analyses.

GENELAND group	predicted group association [%]			
	A	B	C	D
**A**	50.0	33.3	16.7	0.0
**B**	0.0	61.5	23.1	15.4
**C**	0.0	0.0	100.0	0.0
**D**	0.0	0.0	0.0	100.0

65.3% of populations were clustered correctly by the parameter "longitude".
