# Supplementary material for: Widely distributed and regionally isolated! Drivers of genetic structure in Gammarus fossarum in a human-impacted landscape
Source: BMC Evol Biol. 2016 Jul 29;16:153. doi: 10.1186/s12862-016-0723-z (PMC4966747; doi:10.1186/s12862-016-0723-z)
Supplement: Additional file 6: — Pairwise CO1 F ST values between all sampling sites. Sampling sites are indicated by the site abbreviations, and letters from A–G indicate GENELAND groups. Colors indicate within-group F ST values. Asterisks at site names indicate populations where microsatellite loci were also analyzed. Red and bold F ST values indicate significant values. Significance levels were adjusted according to the FDR. (PDF 67 kb) [file 12862_2016_723_MOESM6_ESM.pdf]

Additional file 6: Pairwise CO1 F_{ST} values between all sampling sites. Sampling sites are indicated by the site abbreviations, and letters from A–G indicate GENELAND groups. Colors indicate within-group F_{ST} values. Asterisks at site names indicate populations where microsatellite loci were analyzed. Red and bold F_{ST} values indicate significant values. Significance levels were adjusted according to the FDR.

		A																								B													C						D						E			F
	site	AA	AL*	QB12	E11	E06*	E02*	GS	QB24	QB10	QB17	QB23	VR7	QB22	LE*	LO	ME	KL6	VR17	RO1	RO2	KL9	SB*	QB27	KL3	BB	HSK10	E04	NL	HSK6b	PL1*	PL2	NG*	KL13	SO	VR2	VA	VR5	HB1	HB2	ND	NH*	RU3*	RU4	VR16	VR12	VR23	GB*	NB	VR11	KL14	QB29	E01	KL2
	AA
	AL*	0.21
	QB12	0.09	0.05
	E11	0.10	0.01	0.00
	E06*	0.03	0.15	0.03	0.03
	E02*	0.23	0.00	0.03	0.00	0.13
	GS	0.21	0.00	0.05	0.01	0.15	0.00
	QB24	0.28	0.00	0.07	0.04	0.21	0.00	0.00
	QB10	0.08	0.02	0.00	0.00	0.01	0.01	0.02	0.05
	QB17	0.24	0.00	0.06	0.02	0.17	0.00	0.00	0.00	0.03
	QB23	0.24	0.00	0.06	0.02	0.17	0.00	0.00	0.00	0.03	0.00
A	VR7	0.28	0.00	0.07	0.04	0.21	0.00	0.00	0.00	0.05	0.00	0.00
	QB22	0.32	0.00	0.09	0.05	0.24	0.00	0.00	0.00	0.06	0.00	0.00	0.00
	LE*	0.08	0.04	0.00	0.00	0.02	0.02	0.04	0.06	0.00	0.05	0.05	0.06	0.07
	LO	0.21	0.00	0.05	0.01	0.15	0.00	0.00	0.00	0.02	0.00	0.00	0.00	0.00	0.04
	ME	0.04	0.03	0.00	0.00	0.02	0.01	0.03	0.06	0.00	0.04	0.04	0.06	0.08	0.00	0.03
	KL6	0.33	0.00	0.09	0.05	0.25	0.00	0.00	0.00	0.06	0.00	0.00	0.00	0.00	0.07	0.00	0.08
	VR17	0.06	0.18	0.06	0.09	0.07	0.19	0.18	0.22	0.08	0.19	0.19	0.22	0.24	0.06	0.18	0.10	0.25
	RO1	0.16	0.00	0.03	0.00	0.10	0.00	0.00	0.00	0.01	0.00	0.00	0.00	0.00	0.02	0.00	0.01	0.00	0.15
	RO2	0.07	0.05	0.01	0.00	0.02	0.04	0.05	0.09	0.00	0.06	0.06	0.09	0.10	0.01	0.05	0.00	0.10	0.10	0.03
	KL9	0.32	0.00	0.09	0.05	0.25	0.00	0.00	0.00	0.06	0.00	0.00	0.00	0.00	0.07	0.00	0.08	0.00	0.24	0.00	0.10
	SB*	0.15	0.01	0.02	0.00	0.05	0.00	0.01	0.02	0.00	0.01	0.01	0.02	0.02	0.02	0.01	0.00	0.02	0.16	0.00	0.01	0.02
	QB27	0.32	0.00	0.09	0.05	0.24	0.00	0.00	0.00	0.06	0.00	0.00	0.00	0.00	0.07	0.00	0.08	0.00	0.24	0.00	0.10	0.00	0.02
	KL3	0.03	0.10	0.05	0.05	0.02	0.11	0.10	0.12	0.04	0.11	0.11	0.12	0.13	0.05	0.10	0.04	0.13	0.07	0.08	0.02	0.13	0.08	0.13
	BB	0.85	1.00	0.91	0.96	0.91	0.98	1.00	1.00	0.93	1.00	1.00	1.00	1.00	0.88	1.00	0.96	1.00	0.71	1.00	0.93	1.00	0.93	1.00	0.75
	HSK10	0.77	1.00	0.87	0.94	0.86	0.97	1.00	1.00	0.90	1.00	1.00	1.00	1.00	0.85	1.00	0.93	1.00	0.64	1.00	0.90	1.00	0.92	1.00	0.70	0.00
	E04	0.85	1.00	0.91	0.96	0.91	0.98	1.00	1.00	0.93	1.00	1.00	1.00	1.00	0.88	1.00	0.96	1.00	0.71	1.00	0.93	1.00	0.93	1.00	0.75	0.00	0.00
	NL	0.68	0.95	0.81	0.87	0.77	0.93	0.95	0.96	0.83	0.95	0.95	0.96	0.97	0.80	0.95	0.85	0.97	0.56	0.93	0.83	0.97	0.88	0.97	0.65	0.08	0.00	0.08
	HSK6b	0.44	0.70	0.57	0.59	0.51	0.73	0.70	0.76	0.58	0.72	0.72	0.76	0.78	0.58	0.70	0.58	0.78	0.32	0.65	0.58	0.78	0.68	0.78	0.47	0.29	0.20	0.29	0.07
	PL1*	0.23	0.49	0.33	0.35	0.28	0.53	0.49	0.56	0.34	0.51	0.51	0.56	0.59	0.34	0.49	0.35	0.60	0.09	0.43	0.35	0.60	0.47	0.59	0.26	0.52	0.41	0.52	0.29	0.05
B	PL2	0.90	0.98	0.93	0.96	0.94	0.97	0.98	0.98	0.94	0.98	0.98	0.98	0.99	0.91	0.98	0.96	0.99	0.79	0.98	0.94	0.99	0.94	0.99	0.81	0.00	0.00	0.00	0.09	0.42	0.65
	NG*	0.40	0.60	0.53	0.53	0.45	0.64	0.60	0.65	0.52	0.62	0.62	0.65	0.67	0.54	0.60	0.51	0.68	0.42	0.56	0.51	0.68	0.60	0.67	0.43	0.72	0.66	0.72	0.61	0.49	0.40	0.80
	KL13	0.26	0.42	0.36	0.36	0.30	0.46	0.42	0.47	0.35	0.44	0.44	0.47	0.49	0.37	0.42	0.35	0.49	0.24	0.39	0.35	0.49	0.44	0.49	0.30	0.47	0.41	0.47	0.35	0.23	0.17	0.57	0.35
	SO	0.54	0.84	0.69	0.73	0.63	0.85	0.84	0.89	0.71	0.86	0.86	0.89	0.90	0.69	0.84	0.72	0.90	0.42	0.81	0.71	0.90	0.79	0.90	0.55	0.26	0.14	0.26	0.00	0.00	0.13	0.36	0.53	0.26
	VR2	0.79	0.91	0.84	0.87	0.83	0.91	0.91	0.93	0.85	0.92	0.92	0.93	0.93	0.83	0.91	0.87	0.93	0.68	0.90	0.86	0.93	0.88	0.93	0.73	0.01	0.00	0.01	0.00	0.23	0.47	0.02	0.70	0.46	0.13
	VA1	0.63	0.87	0.76	0.79	0.71	0.88	0.87	0.90	0.77	0.89	0.89	0.90	0.92	0.75	0.87	0.78	0.92	0.52	0.85	0.77	0.92	0.83	0.92	0.62	0.10	0.03	0.10	0.00	0.05	0.25	0.16	0.57	0.33	0.00	0.02
	VR5	0.60	0.77	0.68	0.70	0.65	0.79	0.77	0.81	0.69	0.79	0.79	0.81	0.82	0.69	0.77	0.70	0.83	0.48	0.75	0.70	0.83	0.76	0.82	0.59	0.13	0.08	0.13	0.00	0.01	0.21	0.19	0.58	0.33	0.00	0.09	0.00
	HB1	0.68	0.96	0.85	0.89	0.82	0.94	0.96	0.97	0.86	0.96	0.96	0.97	0.97	0.84	0.96	0.88	0.97	0.69	0.94	0.86	0.97	0.89	0.97	0.69	0.95	0.92	0.95	0.85	0.67	0.64	0.96	0.64	0.50	0.75	0.86	0.78	0.73
	HB2	0.77	1.00	0.90	0.95	0.89	0.97	1.00	1.00	0.92	1.00	1.00	1.00	1.00	0.88	1.00	0.94	1.00	0.74	1.00	0.92	1.00	0.93	1.00	0.73	1.00	1.00	1.00	0.94	0.76	0.72	0.98	0.69	0.55	0.85	0.90	0.86	0.79	0.01
C	ND	0.22	0.60	0.50	0.50	0.39	0.65	0.60	0.67	0.48	0.63	0.63	0.67	0.70	0.51	0.60	0.44	0.71	0.36	0.54	0.47	0.70	0.59	0.70	0.37	0.75	0.66	0.75	0.59	0.45	0.34	0.84	0.43	0.30	0.49	0.72	0.57	0.57	0.24	0.33
	NH*	0.59	0.80	0.75	0.76	0.70	0.82	0.80	0.83	0.74	0.81	0.81	0.83	0.84	0.75	0.80	0.73	0.84	0.65	0.78	0.74	0.84	0.79	0.84	0.64	0.81	0.77	0.81	0.73	0.64	0.61	0.86	0.58	0.50	0.68	0.78	0.70	0.69	0.02	0.06	0.19
	RU3*	0.29	0.59	0.52	0.52	0.45	0.63	0.59	0.64	0.51	0.61	0.61	0.64	0.66	0.54	0.59	0.48	0.66	0.42	0.55	0.51	0.66	0.59	0.66	0.43	0.72	0.67	0.72	0.61	0.49	0.41	0.80	0.47	0.36	0.54	0.70	0.59	0.58	0.18	0.24	0.00	0.14
	RU4	0.67	1.00	0.87	0.93	0.83	0.97	1.00	1.00	0.89	1.00	1.00	1.00	1.00	0.85	1.00	0.91	1.00	0.69	1.00	0.88	1.00	0.91	1.00	0.69	1.00	1.00	1.00	0.89	0.68	0.63	0.98	0.63	0.50	0.76	0.88	0.80	0.75	0.00	0.00	0.22	0.00	0.17
	VR16	0.79	0.94	0.87	0.90	0.85	0.93	0.94	0.95	0.87	0.95	0.95	0.95	0.96	0.85	0.94	0.89	0.96	0.74	0.93	0.87	0.96	0.90	0.96	0.72	0.94	0.92	0.94	0.88	0.74	0.71	0.95	0.69	0.56	0.81	0.87	0.83	0.78	0.89	0.93	0.71	0.77	0.69	0.91
	VR12	0.79	0.93	0.86	0.89	0.84	0.92	0.93	0.94	0.87	0.93	0.93	0.94	0.94	0.85	0.93	0.88	0.95	0.75	0.92	0.87	0.95	0.89	0.94	0.73	0.92	0.90	0.92	0.87	0.76	0.73	0.94	0.70	0.57	0.81	0.87	0.83	0.78	0.88	0.92	0.72	0.78	0.70	0.90	0.00
D	VR23	0.81	0.93	0.88	0.90	0.86	0.93	0.93	0.94	0.88	0.94	0.94	0.94	0.95	0.86	0.93	0.89	0.95	0.77	0.93	0.88	0.95	0.90	0.95	0.74	0.93	0.92	0.93	0.88	0.78	0.75	0.94	0.72	0.59	0.84	0.88	0.85	0.80	0.89	0.93	0.75	0.79	0.72	0.91	0.00	0.00
	GB*	0.80	0.97	0.88	0.92	0.87	0.95	0.97	0.97	0.89	0.97	0.97	0.97	0.98	0.86	0.97	0.91	0.98	0.74	0.96	0.89	0.98	0.91	0.98	0.72	0.97	0.95	0.97	0.90	0.75	0.72	0.96	0.69	0.56	0.83	0.89	0.84	0.79	0.91	0.96	0.71	0.78	0.69	0.94	0.00	0.00	0.00
	NB	0.87	1.00	0.92	0.97	0.93	0.98	1.00	1.00	0.94	1.00	1.00	1.00	1.00	0.90	1.00	0.96	1.00	0.79	1.00	0.94	1.00	0.94	1.00	0.76	1.00	1.00	1.00	0.96	0.81	0.78	0.98	0.74	0.60	0.90	0.92	0.90	0.83	0.96	1.00	0.78	0.82	0.74	1.00	0.02	0.02	0.02	0.01
	VR11	0.85	0.97	0.91	0.94	0.90	0.96	0.97	0.98	0.92	0.98	0.98	0.98	0.98	0.89	0.97	0.94	0.98	0.79	0.97	0.92	0.98	0.93	0.98	0.76	0.97	0.97	0.97	0.93	0.81	0.78	0.97	0.74	0.60	0.88	0.91	0.88	0.82	0.94	0.97	0.77	0.82	0.74	0.96	0.00	0.01	0.01	0.00	0.00
	KL14	0.57	0.73	0.67	0.68	0.62	0.75	0.73	0.76	0.67	0.74	0.74	0.76	0.78	0.68	0.73	0.66	0.78	0.57	0.70	0.66	0.78	0.72	0.78	0.57	0.76	0.72	0.76	0.68	0.58	0.54	0.83	0.56	0.45	0.62	0.74	0.66	0.65	0.70	0.74	0.54	0.66	0.56	0.70	0.74	0.74	0.76	0.74	0.78	0.78
E	QB29	0.64	0.80	0.74	0.75	0.69	0.82	0.80	0.84	0.74	0.82	0.82	0.84	0.85	0.74	0.80	0.74	0.85	0.63	0.78	0.71	0.85	0.79	0.85	0.59	0.81	0.77	0.81	0.73	0.63	0.59	0.87	0.60	0.48	0.67	0.78	0.70	0.68	0.75	0.79	0.59	0.69	0.60	0.75	0.78	0.78	0.80	0.79	0.83	0.82	0.65
	E01	0.62	0.90	0.78	0.81	0.71	0.90	0.90	0.93	0.78	0.91	0.91	0.93	0.94	0.77	0.90	0.79	0.94	0.61	0.87	0.78	0.94	0.84	0.94	0.61	0.91	0.86	0.91	0.78	0.61	0.55	0.94	0.57	0.44	0.68	0.83	0.72	0.69	0.81	0.89	0.55	0.71	0.58	0.83	0.84	0.84	0.86	0.86	0.93	0.90	0.65	0.70
F	KL2	0.90	1.00	0.94	0.97	0.95	0.98	1.00	1.00	0.95	1.00	1.00	1.00	1.00	0.91	1.00	0.97	1.00	0.82	1.00	0.95	1.00	0.95	1.00	0.79	1.00	1.00	1.00	0.97	0.85	0.83	0.99	0.78	0.64	0.92	0.93	0.92	0.86	0.97	1.00	0.82	0.85	0.78	1.00	0.96	0.94	0.95	0.98	1.00	0.98	0.81	0.86	0.95
G	KL15	0.90	1.00	0.94	0.98	0.95	0.98	1.00	1.00	0.95	1.00	1.00	1.00	1.00	0.92	1.00	0.97	1.00	0.83	1.00	0.95	1.00	0.95	1.00	0.79	1.00	1.00	1.00	0.97	0.85	0.83	0.99	0.78	0.65	0.93	0.93	0.93	0.86	0.97	1.00	0.83	0.85	0.78	1.00	0.96	0.95	0.95	0.98	1.00	0.98	0.81	0.86	0.95	1.00
